# Fluorinated Paramagnetic Complexes: Sensitive and Responsive Probes for Magnetic Resonance Spectroscopy and Imaging

**DOI:** 10.3389/fchem.2018.00160

**Published:** 2018-05-23

**Authors:** Katie L. Peterson, Kriti Srivastava, Valérie C. Pierre

**Affiliations:** ^1^Department of Chemistry, Bemidji State University, Bemidji, MN, United States; ^2^Department of Chemistry, University of Minnesota, Minneapolis, MN, United States

**Keywords:** fluorine, lanthanide, iron, magnetic resonance imaging, magnetic resonance spectroscopy, contrast agent, molecular probe, responsive probe

## Abstract

Fluorine magnetic resonance spectroscopy (MRS) and magnetic resonance imaging (MRI) of chemical and physiological processes is becoming more widespread. The strength of this technique comes from the negligible background signal in *in vivo* ^19^F MRI and the large chemical shift window of ^19^F that enables it to image concomitantly more than one marker. These same advantages have also been successfully exploited in the design of responsive ^19^F probes. Part of the recent growth of this technique can be attributed to novel designs of ^19^F probes with improved imaging parameters due to the incorporation of paramagnetic metal ions. In this review, we provide a description of the theories and strategies that have been employed successfully to improve the sensitivity of ^19^F probes with paramagnetic metal ions. The Bloch-Wangsness-Redfield theory accurately predicts how molecular parameters such as internuclear distance, geometry, rotational correlation times, as well as the nature, oxidation state, and spin state of the metal ion affect the sensitivity of the fluorine-based probes. The principles governing the design of responsive ^19^F probes are subsequently described in a “how to” guide format. Examples of such probes and their advantages and disadvantages are highlighted through a synopsis of the literature.

## Fluorine MRS and MRI

Fluorine magnetic resonance imaging (MRI) was first reported in 1977, just four years after the development of ^1^H MRI, by Holland who acquired phantom images of NaF and perfluorotributylamine (Holland et al., 1977). In 1985, McFarland obtained the first ^19^F *in vivo* MR images of a rat using a fluorinated probe which accumulates in the liver, Fluosol-DA (McFarland et al., 1985). These pioneering studies demonstrated the key advantages of fluorine nuclei for magnetic resonance spectroscopy (MRS) and imaging (MRI). Today, these advantages are still exploited for monitoring a variety of biological analytes and processes.

The fluorine nuclei (^19^F, *I* = $\frac{1}{2}$) is attractive due to its 100% abundance and high receptivity (83% that of ^1^H). These attributes make it comparable to the ^1^H nuclei (Knight et al., 2011; Ruiz-Cabello et al., 2011). Further, the similar gyromagnetic ratios (γ) of ^19^F and ^1^H allow images to be collected on broadband ^1^H MRI scanners provided broadband amplifiers and dedicated radiofrequency coils are employed to accommodate the slower Larmor frequency of ^19^F (Stares et al., 2018). The primary advantage of ^19^F probes over ^1^H MRI contrast agents is that the background signal in ^19^F MRI is negligible. The body contains low amounts of fluorine that are primarily embedded in the solid matrices of bones and teeth. As a result, those fluorines have very short transverse relaxation times (*T*_2_) and very broad signals that are easily removed with appropriate pulse sequences (Yu et al., 2013a). On the other hand, *T*_1_ (longitudinal relaxation times) and *T*_2_-based ^1^H MRI contrast agents, which are primarily gadolinium- and iron oxide nanoparticle-based, generate contrast by modulating the relaxation rates of the naturally-occurring water molecules. The significant background from the endogenous water can render certain imaging more difficult. In addition, ^19^F nuclei have a large chemical shift range, >300 ppm, that readily allows for the design of fluorine probes featuring two distinct resonances that can be independently imaged if they are sufficiently separated. As will be discussed later, this property is advantageous in the design of ratiometric responsive ^19^F MRI probes. Ratiometric probes can independently map the distribution of the probe and target analyte *in vivo* and enable tracking of different cell types or monitoring of multiple markers simultaneously.

## Improving the sensitivity of ^19^F probes

Despite the advantages of ^19^F MRS and MRI, this field is limited by its low sensitivity that requires the use of high concentrations of probe, typically between 10 and 50 mM. In comparison, Gd-based *T*_1_ contrast agents can be readily detected *in vivo* at substantially lower concentrations (0.1 μM) (Helm et al., 2018). This low sensitivity is due in part to the fact that only the ^19^F nuclei of the probe are detected. Those nuclei are inherently less concentrated than the ^1^H of H_2_O used in ^1^H MRI, hence the lower sensitivity. This issue is usually addressed by increasing the local concentration of ^19^F nuclei. A second underlying problem of ^19^F probes, and particularly diamagnetic ones, is the long *T*_1_ relaxation times of ^19^F, typically 0.5–3 s for small diamagnetic compounds, which necessitate long image acquisition times in order to obtain sufficient signal-to-noise ratio (SNR). This issue is best addressed via the incorporation of appropriate paramagnetic metal ions that shorten the relaxation rates of the ^19^F nuclei.

### Increasing sensitivity by increasing the density of ^19^F nuclei

The most straightforward approach to increasing ^19^F MRS/MRI signal intensity is simply to increase the local concentration of ^19^F nuclei. This is most often accomplished by increasing the number of ^19^F nuclei on the probe. Complications of this approach are attributed to the hydrophobicity of fluorine which decreases the solubility of a probe in water and affects biodistribution and clearance. Nonetheless, many perfluorocarbons have successfully been used as ^19^F MRS/MRI oximetry sensors and ^19^F cell tracking agents provided they are injected as stable emulsions formulated to optimize clearance (Janjic and Ahrens, 2009; Ruiz-Cabello et al., 2011). Highly fluorinated molecules are mostly inert and considered non-toxic, which has facilitated their use *in vivo* and translation to humans. For instance, fluorinated nano-emulsions are undergoing a phase I clinical trial for ^19^F MRI cell tracking applications (Ahrens and Zhong, 2013; Ahrens et al., 2014). However, not all fluorinated compounds can be assumed to be entirely non-toxic, especially at the high concentrations required for ^19^F MRS/MRI. For instance, perfluorooctane sulfonate (PFOS) and perfluorooctanoic acid (PFOA) are both known to affect the function of the pancreas, thyroid, and liver (Chang et al., 2014; Kamendulis et al., 2014).

An often overlooked complication of perfluorinated probes arise when their ^19^F nuclei are not chemically equivalent. Non-equivalent ^19^F nuclei of a probe that have small frequency differences between the ^19^F resonances result in blurry MR images that are the result of incomplete overlap of the images resulting from each ^19^F resonance (Janjic et al., 2008). This substantially complicates image acquisition, can lead to artifacts, and decreases SNR. It is thus best to increase the number of fluorines in such a way that they remain chemically equivalent.

**Figure 1:**
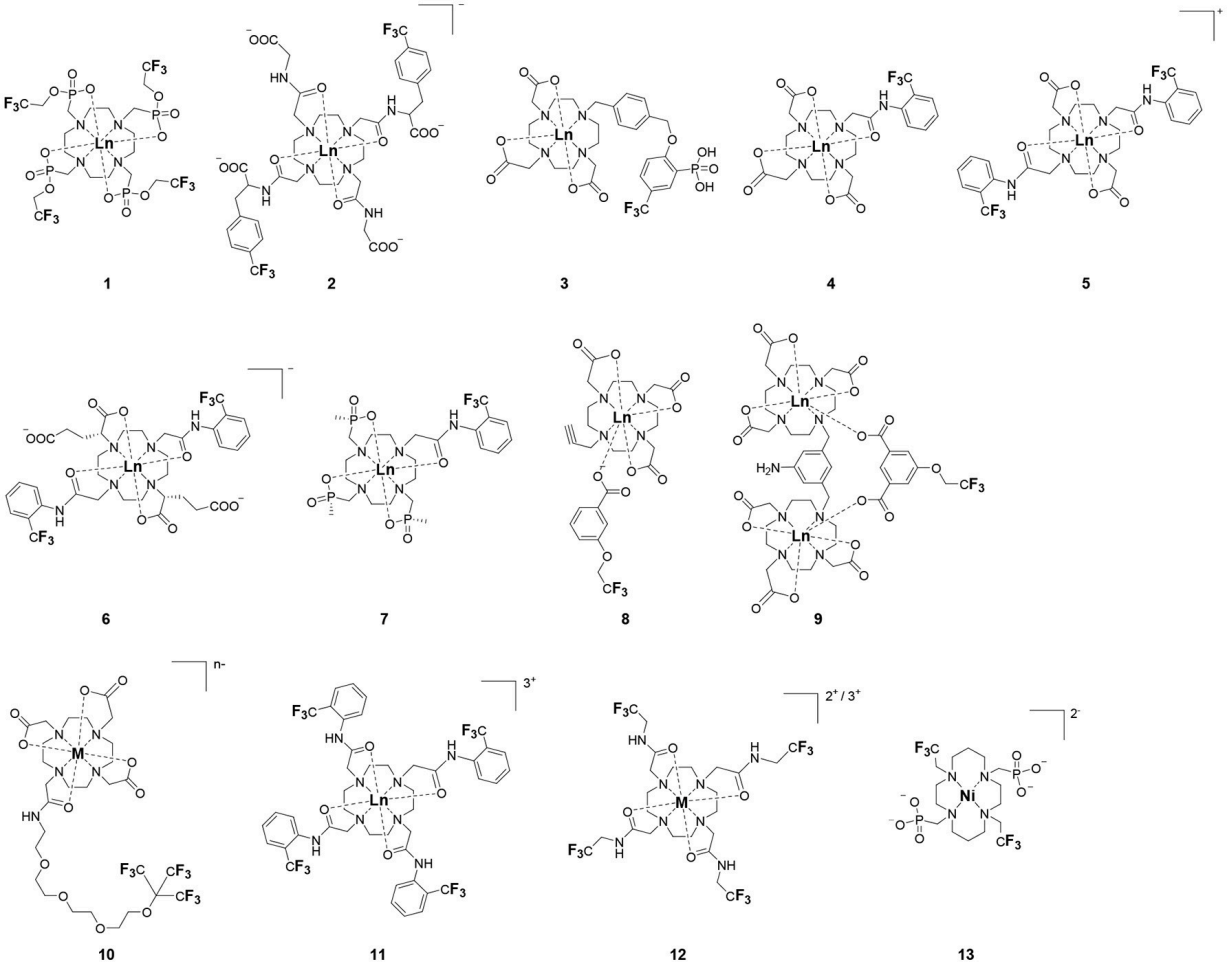
Chemical structures of non-responsive paramagnetic ^19^F probes (**1**, Kim et al., 1997; **2**, Cakic et al., 2016; **3**, Placidi et al., 2013; **4**, Chalmers et al., 2010; **5–6**, Chalmers et al., 2011a; **7**, Chalmers et al., 2011a,b; **8–9**, Davies et al., 2015; **10**, Jiang et al., 2011; **11**, Chalmers et al., 2010; **12**, Srivastava et al., 2017a,b; **13**, Blahut et al., 2016, 2017).

One of the most efficient ways to ensure that each ^19^F nuclei is chemically equivalent is to incorporate high molecular symmetry in the design. Transition metal and lanthanide complexes with C_3_ or C_4_ symmetry are ideally suited for this application if the ligands are fluorinated appropriately. The macrocyclic DOTA (1,4,7,10-tetraazacyclododecane-1,4,7,10-tetraacetic acid) ligand, for instance, adopts a pseudo C_4_ symmetry in which the acetate arms are arranged in a propeller-like fashion above the plane of the coordinating nitrogen atoms. Four possible isomers exist for this system based on the combination of two macrocyclic ring configurations (square prismatic or square antiprismatic) and the two possible arrangements of the acetate arms (Caravan et al., 1999; Benetollo et al., 2003). The substituents on the acetate arms and the Ln^III^ ion both influence the ratio of isomers observed by NMR such that one isomer can be favored over the others (Parker et al., 2002). The judicial choice of the macrocycle's arms has a notable impact on the ^19^F NMR spectra of this class of macrocycles and on their effectiveness to function as ^19^F MRS/MRI probes. Ln(F-DOTPME)^−^ (1,4,7,10,-tetraazacyclododecane-1,4,7,10-tetrakis(methanephosphonic acid mono(2′,2′,2′-trifluoroethyl) ester)), the lanthanide complex of the tetra trifluoroethyl-substituted DOTMP (1,4,7,10-tetraazacyclododecane-1,4,7,10-tetrayl-tetrakis(methylphosphonic acid)) analog (**1**, Figure 1; Kim et al., 1997), for instance, exists in solution at room temperature as a mixture of all eight isomers, which results in the presence of eight distinct ^19^F peaks within a 12 (Yb^III^) to 70 ppm (Tm^III^) range. As discussed above, the presence of these different isomers limits the efficacy of these complexes to function as ^19^F MR probes. The metal complexes of the tetra substituted DOTA with aryl-CF_3_ groups (**11**, Figure 1; Chalmers et al., 2010) exist primarily as one isomer, whose proportion depends on the nature of the lanthanide ion. This ligand is therefore better suited for ^19^F MRI applications. The problem of multiple isomers is not limited to tetra-substituted macrocyclic derivatives. In fact, many DOTA analogs with only one or two fluorinated arms are present in solution as more than one isomer (**2**–**4**), the percentage of which varies according to the lanthanide (Kenwright et al., 2008; Chalmers et al., 2010; Placidi et al., 2013; Cakic et al., 2016).

Substitution of one or more carboxylate arms with bulky phosphinate groups or the introduction of substituent at the α-position to the ring nitrogen affects this dynamic process. For instance, Chalmers et al. reported two DOTA derivatives incorporating two trifluoroaryl groups and either two carboxylic acid or two glutaric acid substituents at the α-position to the ring nitrogen (**5** and **6**, Figure 1) (Chalmers et al., 2011a,b). In solution the carboxylic acid derivative, unlike the glutamic acid one, favors one major isomer which accounts for 80% of the ^19^F NMR spectrum. The selectivity for one isomer can also be improved *via* the use of a phosphinate arm. For instance, all lanthanide complexes of **7** exist primarily (>87%) as one isomer. In extreme cases, a single isomer of a DOTA analog fluorinated on a single arm can be obtained, as for compounds **8**–**10** (Figure 1) (Jiang et al., 2011; Davies et al., 2015).

A single isomer for a tetra-substituted DOTA complex which exploits the symmetry of the ligand in order to maximize the number of equivalent fluorines can nonetheless be obtained. Examples include M-DOTAm-F12 (2,2′,2″,2^‴^-(1,4,7,10-tetraazacyclododecane-1,4,7,10-tetrayl)tetrakis(N-(2,2,2-trifluoroethyl)acetamide)) (12, Figure 1; Srivastava et al., 2017a,b) and more recently the redox-responsive Eu-complex **39** (Figure 9) (Basal et al., 2017). Both of these complexes contain 12 ^19^F nuclei linked to the macrocycle via four amide arms. Importantly, they both exist as a single isomer for which all 12 ^19^F nuclei remain chemically equivalent. This increases the sensitivity of the ^19^F MR probe and the resolution of the images that can be obtained with it. The high symmetry of the DOTA scaffold can also be exploited with certain transition metals. The unusual but stable 8-coordinate structure of the Fe^II^-DOTAm-F12 maintains a high symmetry and chemical equivalency for its 12 fluorine nuclei (Srivastava et al., 2017a). The higher solubility in water of the M-DOTAm-F12, >100 mM, renders it particularly promising for further development and imaging applications. In summary, the C_3_ or C_4_ symmetry of metal complexes can be exploited to increase the number of chemically equivalent ^19^F nuclei on a probe, but the drawback of multiple stable isomers in solution remains a challenge.

### Increasing sensitivity with paramagnetic metal ions

Increasing the number of ^19^F nuclei per agent can only go so far in terms of increasing the sensitivity of ^19^F probes. Ultimately, there is a limit to the number of ^19^F nuclei that can be added to a molecule in such a way that they are chemically equivalent all the while maintaining sufficient water solubility for *in vivo* applications. A complementary approach to increasing the number of ^19^F nuclei that is often used simultaneously is to decrease the *T*_1_ of the ^19^F nuclei. Diamagnetic molecules typically have *T*_1_ of ^19^F nuclei in the range of 0.5–3 s. For *in vivo* imaging applications, that are limited by time, the long *T*_1_ of diamagnetic fluorine probes hinders their sensitivity (Harvey et al., 2012b). It is thus preferential to decrease the *T*_1_ of the ^19^F nuclei of fluorine probes. This is best achieved by incorporating paramagnetic metal ions such as lanthanides(III) or iron(II). The metal should be selected with care as different paramagnetic metal ions have different effects on the relaxation of nearby nuclei.

The effect of a paramagnetic metal ion on the *T*_1_ and *T*_2_ of a ^19^F nuclei is described by the Bloch-Wangsness-Redfield theory. A ^19^F nuclei adjacent to a paramagnetic center in a magnetic field can relax via five different mechanisms: (1) chemical shift anisotropy, (2) inter-nuclear dipole-dipole interaction, (3) electron-nucleus contact interaction, (4) electron-nucleus dipole-dipole interaction, and (5) Curie relaxation (Chalmers et al., 2010). Of these, chemical shift anisotropy is negligible for a single ^19^F nuclei or CF_3_ group, and inter-nuclear dipole-dipole mechanisms are minimal, accounting for <1% of the total relaxation. The electron-nucleus contact interaction is also negligible since ligand geometries generally locate the ^19^F nuclei further than 4.5–7.5 Å away from the metal ion, resulting in coupling values of zero. Thus, only the electron-nucleus dipole-dipole and Curie relaxation processes contribute significantly to the relaxation of the ^19^F nuclei. Given this, the longitudinal (*R*_1_ = 1/*T*_1_) and transverse (*R*_2_ = 1/*T*_2_) relaxation rates of ^19^F nuclei in paramagnetic metal complexes are described by Equations (1) and (2), respectively (Chalmers et al., 2010).

(1) $$\begin{array}{l} R_{1}=\frac{2}{15}{\left(\frac{\mu_{0}}{4\pi }\right)}^{2}\;\frac{\gamma_{F}^{2}\mu_{\text{eff}}^{2}}{d^{6}}\;\left(\frac{7\tau_{R+e}}{1+\;\omega_{e}^{2}\tau_{R+e}^{2}}+\frac{3\tau_{R+e}}{1+\;\omega_{F}^{2}\tau_{R+e}^{2}}\right)\\ \;+\;\frac{2}{5}{\left(\frac{\mu_{0}}{4\pi }\right)}^{2}\frac{\omega_{F}^{2}\mu_{\text{eff}}^{4}}{{\left(3kT\right)}^{2}d^{6}}\;\frac{{3\tau }_{R}}{1+\;\omega_{F}^{2}\tau_{R}^{2}} \end{array}$$

(2) $$\begin{array}{l} R_{2}=\frac{1}{15}{\left(\frac{\mu_{0}}{4\pi }\right)}^{2}\;\frac{\gamma_{F}^{2}\mu_{\text{eff}}^{2}}{d^{6}}\;\left({4\tau }_{R+e}+\frac{3\tau_{R+e}}{1+\;\omega_{e}^{2}\tau_{R+e}^{2}}+\frac{13\tau_{R+e}}{1+\;\omega_{F}^{2}\tau_{R+e}^{2}}\right)\\ \;+\;\frac{1}{5}{\left(\frac{\mu_{0}}{4\pi }\right)}^{2}\frac{\omega_{F}^{2}\mu_{\text{eff}}^{4}}{{\left(3kT\right)}^{2}d^{6}}\;\left({4\tau }_{R}+\frac{{3\tau }_{R}}{1+\;\omega_{F}^{2}\tau_{R}^{2}}\right) \end{array}$$

where

(3) $$\begin{array}{rcl} \mu_{\text{eff}}^{2} & = & \;g_{J}^{2}\;\mu_{B}^{2}\;J\left(J+1\right) \end{array}$$

(4) $$\begin{array}{rcl} \omega_{e} & = & \;\left(\frac{g_{J}\mu_{B}}{\hbar }\right)B_{0} \end{array}$$

(5) $$\begin{array}{rcl} \tau_{\text{R}+\text{e}} & = & \left(\tau_{\text{R}}^{-1}+T_{1e}^{-1}\right) \end{array}$$

In these equations, *d* is the distance separating the ^19^F nuclei from the paramagnetic metal ion, μ_0_ is the permeability of vacuum, γ_F_ is the gyromagnetic ratio of the ^19^F nuclei, *T* is the temperature in K, *k* is Boltzmann's constant, and ω_F_ is the Larmor angular frequency of ^19^F. The effective magnetic moment, μ_eff_, is proportional to the effective electron g-factor (*g*_*J*_), the Bohr magneton (μ_B_), and the electron angular momentum, *J(J*+*1)* according to Equation (3). The electron angular frequency (ω_*e*_) is a function of the magnetic field strength (*B*_0_) according to Equation (4) in which ℏ is the reduced Planck's constant. The τ_R+e_ term is dependent on the rotational correlation time (τ_R_) and the electron spin longitudinal relaxation time (*T*_1e_) according to Equation (5).

In Equations (1) and (2), the first term represents the dipolar relaxation characterized by the stochastic electron magnetization of the electron-nucleus dipole-dipole interaction. The second term is the Curie relaxation arising from the interaction between the fluorine nuclear spin and the magnetic dipole induced by the applied magnetic field. Together, they relate *R*_1_ and *R*_2_ to the effective magnetic moment (μ_eff_) of the paramagnetic metal, the ^19^F–metal distance (*d*), the rotational correlation time (τ_R_), the applied magnetic field strength (*B*_0_), and the temperature (*T*). Optimizing the sensitivity of ^19^F MRS/MRI probes requires understanding each of these relationships.

#### Dependence on the nature of the metal ion

The effective magnetic moment (μ_eff_) of the paramagnetic metal affects both the electron-nucleus dipole-dipole interaction and, to a greater extent, the Curie relaxation mechanism. The effective magnetic moment of transition metal complexes is a function of both their oxidation state and their ligand fields. Each lanthanide ion has a characteristic μ_eff_ value (Table 1).

**Table 1:** Magnetic and relaxation properties of lanthanide(III) ions.

**Lanthanide ion**	**Ground state term**	**μ_eff_ (B.M.)^a^**	**μ_eff_ (exp)^b^**	**Bleany Constant (*C*_j_)^c^**	**Electron relaxation time (*T*_1e_)/10^−13^ s^d^**
Ce^III^	^2^F_5/2_	2.56	2.55	−6.3	0.90
Pr^III^	^3^H_4_	3.62	3.47	−11.0	0.57
Nd^III^	^4^I_9/2_	3.68	3.69	−4.2	1.15
Pm^III^	^5^I_4_	2.68	2.41	2.0	Unknown
Sm^III^	^6^H_5/2_	1.55–1.65	1.58	−0.7	0.45
Eu^III^	^7^F_0_	3.40–3.51	3.4	4.0	0.09
Eu^II^	^8^S_7/2_	7.6–8.0		0	10^4^ *^e^*
Gd^III^	^8^S_7/2_	7.94	7.63	0	10^4^-10^5^ *^f^*
Tb^III^	^7^F_6_	9.7	9.8	−86	2.03
Dy^III^	^6^H_15/2_	10.6	10.3	−100	2.99
Ho^III^	^5^I_8_	10.6	10.4	−39	1.94
Er^III^	^4^H_15/2_	9.6	9.4	33	2.38
Tm^III^	^3^H_6_	7.6	7.6	53	3.69
Yb^III^	^2^F_7/2_	4.5	4.3	22	1.37

a *(Gysling and Tsutsui, 1971; Tilley et al., 1980; Garcia and Allen, 2012)*.

b *(Gysling and Tsutsui, 1971; Bertini et al., 1993a; Chalmers et al., 2010)*.

c *(Bleaney, 1972)*.

d *aqua ion, 2.1 T (Alsaadi et al., 1980)*.

e *value for Eu^II^ was calculated using formula in Burai et al. (2003)*.

f *Bertini et al. (1993b)*.

The effect of the nature of the transition or lanthanide ion on the relaxation rates of the ^19^F nuclei of fluorinated complexes and the sensitivity of the resulting fluorine probe has been explored via a series of non-responsive probes. Several fluorinated ligands have been investigated, which enables a more in-depth evaluation of the influence of the structure of the ligand on the ^19^F relaxation rates of the probe (Figure 1, Table 2). Studies by Parker and coworkers focused on a DOPA ([(4,7,-di{[hydroxyl(methyl)phosphoryl]methyl}-10-({[2-(trifluoromethyl)phenyl]carbamoyl}methyl)-1,4,7,10-tetraazacyclododecan-1-yl)methyl](methyl)phosphinic acid) ligand monosubstituted with *ortho* aryl-CF_3_ (**7**) and DOTA ligand scaffold tetra substituted with aryl-CF_3_ groups (**11**) (Chalmers et al., 2010, 2011a). In this scaffold, *ortho* substitution of the CF_3_ group lead to a greater decrease in relaxation times due to the shorter distance separating the ^19^F nuclei from the lanthanide ion (Senanayake et al., 2007; Chalmers et al., 2010). In agreement with the Bloch-Wangsness-Redfield theory, this first study demonstrated that paramagnetic lanthanide ions can increase the *R*_1_ by up to two orders of magnitude, with the greatest change observed with those metal ions with the highest μ_eff_, notably, Tb^III^, Dy^III^, Ho^III^, and Er^III^ (Table 2). Unfortunately, the poor solubility of the tetra substituted complexes in water limited further *in vivo* studies.

**Table 2:** ^19^F chemical shift and relaxation properties for non-responsive paramagnetic fluorine probes.

**Ligand (B_0_)**	**Ln^III^**	**δ (ppm)**	***Δδ* (ppm)**	***R*_1_ (Hz)**	***T*_1_ (ms)**	***R*_2_ (Hz)**	***T*_2_ (ms)**	***T*_2_/*T*_1_**
**2** (7.0 T)*^*a*^*	Eu^III^	−61.8	n.d.	1.4	714	18^h^	56	0.078
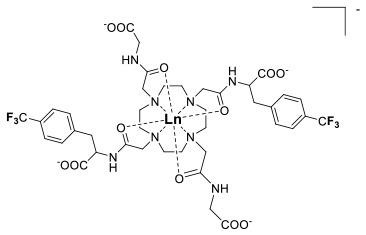		−61.4		1.4	714	19^i^	53	0.074
	Gd^III^	−61.6	n.d.	182	5.5	385	2.6	0.47
	Tb^III^	−65.2	n.d.	32^h^	31	48^h^	21	0.68
		−55.6		12^i^	83	28^i^	36	0.43
**3** (7.0 T)*^*b*^*	Ligand	−61.24	n.d.	n.d.	n.d.	n.d.	n.d.	n.d.
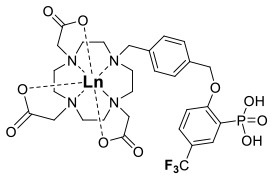	Gd^III^	−60.45	n.d.	322.6	3.1	500	2.0	0.65
	Yb^III^	−60.54	n.d.	3.7	273	238	4.2	0.015
**4** (9.4 T)^c^^*^	Y^III^	−62	0	n.d.	n.d.	n.d.	n.d.	n.d.
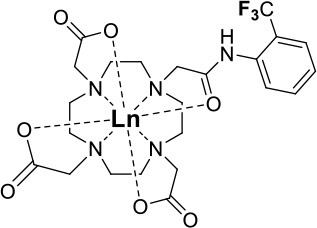	Tb^III^	−51.9	10.1	116	8.62	119	8.4	0.975
	Dy^III^	−64.9	−2.9	139	7.19	220	4.55	0.632
	Ho^III^	−64.2	−2.2	109	9.17	151	6.62	0.722
	Er^III^	−64.8	−2.8	65	15.38	157	6.37	0.414
	Tm^III^	−77.4	−15.4	51	19.61	91	10.99	0.56
**5** (9.4 T)^c*^	Tb^III^	−61.9	n.d.	2.8	357.14	132	7.58	0.021
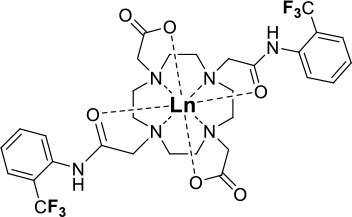	Dy^III^	−99.8	n.d.	158	6.33	213	4.69	0.742
	Ho^III^	−57.7	n.d.	91	10.99	159	6.29	0.572
	Er^III^	−58.6	n.d.	81	12.35	164	6.10	0.494
	Tm^III^	−68.2	n.d.	56	17.86	102	9.80	0.549
**7** (9.4 T)*^*c*^*	Y^III^	−61.2	0			15	66.7	
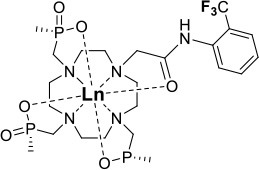	Tb^III^	−47.7	13.5	147	6.8	267	3.75	0.55
	Dy^III^	−63.6	−2.4	185	5.41	251	3.98	0.737
	Ho^III^	−61.5	−0.3	120	8.33	143	6.99	0.839
	Er^III^	−72.6	−11.4	109	9.17	138	7.25	0.79
	Tm^III^	−89.5	−28.3	63	15.87	84	11.9	0.75
**10** (11.7 T)*^*d*^*	ligand	−71.2		0.752	1330	1.43	701	0.53
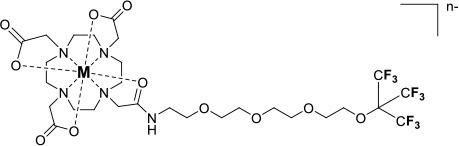	Y^III^	−71.1	0	0.66	1514	1.28	783	0.52
	Gd^III^	−66.6	−4.5	236	4.24	602	1.66	0.39
	Tb^III^	−63.3	−7.8	13.8	72.6	24.0	41.7	0.57
**11** (9.4 T)*^*e*^*	Y^III^	−61.6	0	0.78	1282	2.9	345	0.27
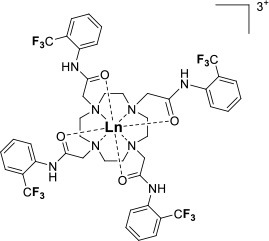	Tb^III^	−53.9	7.7	185	5.41	n.d.	n.d.	n.d.
	Ho^III^	−59	2.6	192	5.21	n.d.	n.d.	n.d.
	Er^III^	−63.5	−1.9	109	9.17	n.d.	n.d.	n.d.
	Tm^III^	−65.1	−3.5	59.7	16.8	n.d.	n.d.	n.d.
**12** (7.0 T)*^*f*^*	ligand	−72.7		1.1	880	1.5	680	0.77
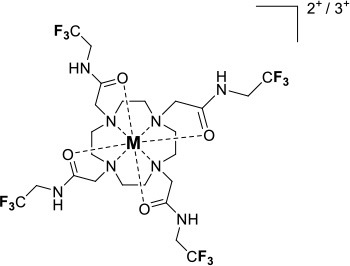	La^III^	−72.1	0	1.8	570	2.5	400	0.70
	Eu^III^	−72.4	−0.3	2.8	360	24	41	0.11
	Gd^III^	−72.0	0.1	83	12	7100	0.14	0.01
	Tb^III^	−54.1	18.0	160	6.3	770	1.3	0.21
	Dy^III^	−52.4	19.7	170	5.9	450	2.2	0.37
	Ho^III^	−61.8	10.3	130	7.6	190	5.4	0.71
	Er^III^	−76.5	−4.4	71	14	110	8.8	0.63
	Tm^III^	−83.3	−11.2	39	26	63	16	0.62
	Yb^III^	−75.9	−3.8	7.7	130	18	55	0.42
	Fe^II^	−70.1	2.0	180	5.7	180	5.6	0.98
**13** (7.0 T)*^*g*^*	ligand	−68.9		2000	0.5	20	50	0.1
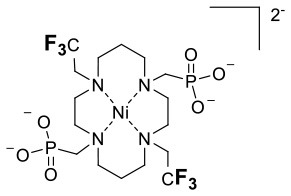	Ni^II^	−26	n.d.	357	2.8	1111	0.9	0.32

* *Data listed for major isomers in solution*.

*(Cakic et al., 2016)*.

*(Placidi et al., 2013)*.

*(Chalmers et al., 2011a)*.

*(Jiang et al., 2011)*.

*(Chalmers et al., 2010)*.

*(Srivastava et al., 2017b)*.

*(Blahut et al., 2016)*.

*Major isomer*.

*Minor isomer*.

Recently, our lab reported the relaxation properties of the Fe^II^ and Ln^III^ complexes of another macrocyclic ligand, DOTAm-F12 (**12**) (Srivastava et al., 2017a,b). Complexes of this series, including the Fe^II^ complex, have a number of advantageous properties for ^19^F imaging. The complexes each contain 12 fluorine nuclei that are chemically equivalent, resulting in a single ^19^F resonance. As discussed above, a high number of fluorine nuclei and their equivalency are two necessary components in the design of sensitive ^19^F probes for MR imaging. The complexes are also highly soluble in water; they can be dissolved in concentrations of up to 100 mM, a necessity for any biological applications. The Fe^II^ complex is notable as it is stable both in air and water and is a rare example of an eight coordinate iron complex (Srivastava et al., 2017a). Importantly, for both the Fe^II^ and the Ln^III^ complexes, the structure of the macrocycle places the fluorine nuclei from 5.22 to 6.84 Å from the metal ion. As will be discussed later, this is an optimum distance since it minimizes the effect of the shortened *T*_2_ of the ^19^F nuclei. As in Parker's study, the paramagnetic metal ions decrease the *T*_1_ of the ^19^F nuclei by up to two orders of magnitude with the greatest decrease observed with those metal ions having the highest μ_eff_, namely Tb^III^, Dy^III^, Ho^III^, and Fe^II^. Paramagnetic Ni^II^ cyclam complexes featuring *trans*-trifluoroethyl groups were also recently reported by Blahut et al. (2016, 2017). The *trans-*[Ni(te2f2p)]^2−^ (**13**, Figure 1) with phosphate ligands has a ^19^F–Ni distance of ~ 5 Å which facilitates a nearly 180-fold reduction in *T*_1_ vs. the free ligand (Table 2). This Ni^II^ complex and Fe^II^-DOTAm-F12 (**12**) exemplify that the diverse electronic and structural parameters of transition metal complexes can also be exploited to optimize ^19^F paramagnetic relaxation enhancement (PRE).

The sensitivity of an agent, estimated by comparing SNR of solutions of complexes at the same concentration, is dependent not only on *T*_1_ but also on *T*_2_. The line-broadening characteristic of the shorter *T*_2_ of the fluorines of certain paramagnetic complexes significantly affect the sensitivity of the probe. An extreme example being the Gd^III^ complex of DOTAm-F12 that has such a short *T*_2_ that the complex could not be observed by MRI. Note that for such complexes with rapid transverse relaxation, the use ultrashort TE (UTE) and zero TE (ZTE) acquisition pulse sequences can sometimes increase the SNR in ^19^F-MRI (Kislukhin et al., 2016).

Not all paramagnetic metals affect *T*_2_ to the same degree as *T*_1_. Therefore, the most sensitive metal-based probes are not those whose metal ions have the highest μ_eff_, but those whose ^19^F have the highest *T*_2_/*T*_1_ ratio. For M-DOTAm-F12 in water, these are Fe^II^, Ho^III^, Tm^III^, and Yb^III^. Importantly, the media also has a substantial effect on the relaxation times, and particularly on the transverse one. The *T*_2_ of all M-DOTAm-F12 complexes decrease substantially in blood. This affects the sensitivity of all metal complexes. Ho^III^-DOTAm-F12, for instance, is the most sensitive probe in water, yet it could not be detected in blood. The lower *T*_2_ value of M-DOTAm-F12 complexes in blood in comparison to water could be due to different parameters. Coordination to albumin or other serum proteins and the resulting increase in rotational correlation time can affect both *T*_1_ and *T*_2_. Blood is also more viscous than water, and this increase in viscosity also affects τ_R_, *T*_1_, and *T*_2_. One should therefore not assume that a probe that is sensitive in water is necessarily appropriate for *in vivo* applications where it will accumulate in blood or tissues. It is therefore recommended that the sensitivity of probes be evaluated not only in water but also in the media in which they are intended to be used. Also note that the greatest effect is not necessarily obtained with lanthanide ions. In blood, the most sensitive probe is Fe^II^-DOTAm-F12 whose limit of detection (300 μM) is more than an order of magnitude lower than that of small diamagnetic probes.

Similar trends have been observed with other lanthanide complexes, such as **2–5**, **7**, and **10–12** (Figure 1, Table 2). In general, the stronger relaxing metals with the higher μ_eff_ near 10, Tb^III^, Dy^III^, and Ho^III^ decrease both *T*_1_ and *T*_2_ the most. Lanthanides such as Er^III^, Tm^III^, and in the extreme case Gd^III^, have lower *T*_2_/*T*_1_ ratio and hence lower sensitivity (Table 2) (Chalmers et al., 2010, 2011a,b; Jiang et al., 2011; Harvey et al., 2012b; Placidi et al., 2013; Blahut et al., 2016; Cakic et al., 2016; Srivastava et al., 2017b). Do note that the PRE effect is determined by other parameters beyond the μ_eff_ of the metal ion; it is also dependent on the magnetic field strength and on the distance separating the ^19^F nuclei and the paramagnetic metal ion (Equation 1–5). As mentioned above, transition metals such as Fe^II^ (μ_eff_ ~ 5) and Ni^II^ (μ_eff_ ~ 3) (complexes **12** and **13**, respectively) can also generate high ^19^F MRI SNR ratios of 20–30 despite their lower magnetic moments.

Even at greater ^19^F–M distances, the effect of the paramagnetic metal ion on the relaxation rates of the fluorine nuclei can be notable. The fluorinated complex of Jiang (**10**, Figure 1) is a DOTA-based complex containing three equivalent trifluoromethyl groups separated by a polyethylene glycol linker that positions the ^19^F nuclei more than 10 Å away from the paramagnetic metal ion. Despite this great distance, paramagnetic metals such as Tb^III^, Dy^III^, Ho^III^, Er^III^, Gd^III^, Fe^III^, Ni^II^, and Cu^II^ reduce *T*_1_ and *T*_2_ by >95% (Jiang et al., 2011). A noteworthy conclusion of this study is that the ^19^F relaxation times of these paramagnetic metal complexes are relatively constant (<5% change) with respect to environmental fluctuations in pH, temperature, and the concentration of either O_2_ or bovine serum albumin (BSA) (Jiang et al., 2011). It is thus the identity of the paramagnetic metal ion that is the primary contributor to both the chemical shift and the relaxation rates of the fluorines. This observation is important for the development of responsive probes (*vide infra*).

#### Dependence on the ^19^F-metal distance

From the Bloch-Wangsness-Redfield Equations (1–5) given above, both the *R*_1_ and the *R*_2_ of the fluorine nuclei have a steep dependence on the distance *d* separating the ^19^F nuclei from the metal ion. The ^19^F–M distance is a key parameter in determining the sensitivity of metal-based fluorine probes. The effect of the paramagnetic metal ion on *R*_2_ extends to longer distances than that of *R*_1_. Both too long and too short a distance can be detrimental. Too long a ^19^F–M distance and the effect of the paramagnetic metal ion on the relaxation times of the fluorine nuclei is severely diminished. Too short a distance, and the substantial shortening of *T*_2_ decreases the sensitivity of the fluorine probe. A higher *T*_2_/*T*_1_ ratio and thus a more sensitive fluorine probe is obtained if the ^19^F nuclei are positioned between 4.5 and 7.5 Å from the metal ion (Harvey et al., 2012b).

Within this range, even minor changes in ^19^F–M ion distance can have an impact on the relaxation rates of fluorine nuclei and the sensitivity of the agent. One such example are the lanthanide complexes containing a single aryl-CF_3_ moiety positioned either on an acetate-based DOTA (**4**, Figure 1) or a phosphinate-based DOPA (**7**, Figure 1) scaffold. The DOTA complexes (**4**) exists in solution as a mixture of multiple isomers with the primary species representing only 50% of the fluorine signal intensity by NMR. On the other hand, the principal isomer of the phosphinate analog (**7**) accounts for 87% of the total ^19^F signal intensity (Chalmers et al., 2011a). The *R*_1_ and *R*_2_ of lanthanide complexes of these two ligands are different. The *R*_1_ of complexes of DOPA scaffold are 10–67% greater than those of the DOTA scaffold (Table 2). However, the *R*_2_ of complexes (Ho^III^, Er^III^, and Tm^III^) of DOPA scaffold are 5–12% smaller than those of DOTA scaffold with an exception of Tb^III^ and Dy^III^ being 14–124% greater than those of DOTA complexes. Global fitting of the relaxation rates collected at multiple field strengths to the Bloch-Wangsness-Redfield theory determined that the differences in *R*_1_ and *R*_2_ are due to a 0.3 Å shorter ^19^F–Ln distance in the DOPA complex than in the DOTA one (Chalmers et al., 2010, 2011a). Interestingly, the highest *T*_2_/*T*_1_ ratio with the DOTA-based scaffold was obtained with Tb^III^. For the DOPA-based scaffold, it was instead Ho^III^ (0.84), Er^III^ (0.79), Tm (0.75), and Dy (0.74) that yielded the highest *T*_2_/*T*_1_ ratio. As is apparent from this example, the best metal ion for one fluorinated ligand is not necessarily the same for another one.

The effect that such minor modifications of the ligand and the structure of the complex have on the *T*_2_/*T*_1_ ratio and the sensitivity of the probe has been observed with other complexes. The Yb^III^ -DO3A (1,4,7,10-tetraazacyclododecane-1,4,7-tris(acetic acid))-based complex with a fluorinated aryl phosphonate of Angelovski, (**3**, Figure 1) positions the ^19^F nuclei 7 Å from the metal, a ^19^F–Yb distance 0.7 Å greater than in Yb-DOTAm-F12 (Placidi et al., 2013). At 7 T, the *T*_1_ of 273 ms is nearly twice that in Yb^III^-DOTAm-F12. More noticeably, the very short *T*_2_ of the fluorines of this complex, 4.2 ms, results in a much smaller *T*_2_/*T*_1_ ratio and hence a much less sensitive probe than Yb^III^-DOTAm-F12.

Supramolecular self-assembly enables more facile synthesis of paramagnetic fluorine probes via the formation of ternary complexes. Given the hardness of lanthanide ions, fluorinated carboxylate ligands are particularly well-suited for this approach (**8** and **9**, Figure 1) (Davies et al., 2015). These assemblies also decrease the relaxation times of the fluorine to a similar degree as observed in the complexes discussed above, although the relatively weak affinity of the carboxylate ligand for the lanthanide (log *K*_a_ ~ 4.9) may result in decomplexation of the fluorinated moiety under biological conditions.

**Figure 2:**
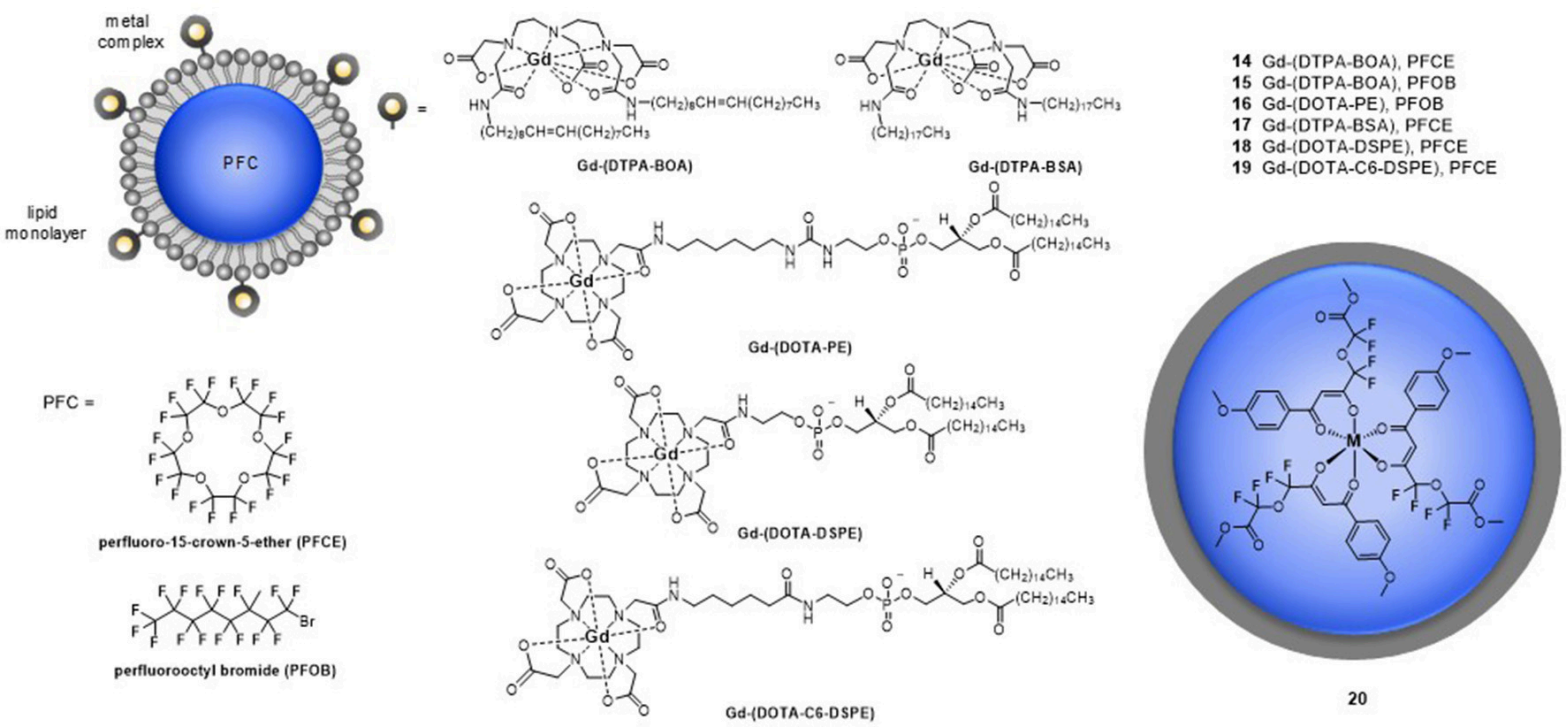
Schematic representations of paramagnetic perfluorocarbon nanoparticles. Gd-complexes are embedded into lipid monolayers that encapsulate perfluorocarbon cores (**14**, Neubauer et al., 2008; **15–16**, Hu et al., 2011; **17–19**, de Vries et al., 2014) or perfluorocarbon substituted parmagnetic metal complexes form nano-emulsions (**20**, Kislukhin et al., 2016).

Similarly, paramagnetic metals have also been employed to increase the relaxation rates of fluorine nuclei in perfluorinated emulsions. Perfluorocarbon-based emulsions present an advantage over monomolecular complexes in that they contain a higher density of fluorine nuclei which increases their sensitivity. The design and application of perfluorocarbon tracers for ^19^F MRI have been reviewed elsewhere. (Knight et al., 2011; Tirotta et al., 2015) Here, the role of Gd^III^ and Fe^III^ in enhancing the relaxation rates of fluorine nuclei in nanoemulsions is explored. Neubauer et al. first reported a paramagnetic fluorinated emulsion containing a liquid perfluoro-15-crown-5 ether core encased in a lipid layer surface-coated with Gd-DTPA-BOA (6,9-bis(carboxymethyl)-3-(2-(((E)-octadec-9-en-1-yl)amino)-2-oxoethyl)-11-oxo-3,6,9,12-tetraazatriacont-21-enoic acid) complexes (**14**, Figure 2) (Neubauer et al., 2008). The relaxation rates of the fluorine nuclei in this paramagnetic emulsion increases according to an outer-sphere mechanism which requires the perfluorocarbons within the core of the nanoparticle to diffuse close to the Gd^III^ ions on the surface of the lipid monolayer. In this 200 nm assembly, the *R*_1_ and *R*_2_ of the fluorine nuclei located ~ 15 Å away from the Gd^III^ ion increase 4- and 8-fold, respectively. This, in turn, increases the sensitivity of the emulsion 2-fold (Neubauer et al., 2008). Further calculations on how the size of the nanoparticles, the ^19^F–Gd distance (*d*), and the diffusion coefficient of the perfluorocarbon affect the *R*_1_ and *R*_2_ of the ^19^F enabled further improvement of the assembly (Hu et al., 2011). A 4-fold relaxation enhancement was observed with a perfluoro-15-crown-5 ether and Gd-DTPA-BOA (**14**) compared to non-metallated perfluorocarbon. Additionally, a shorter linker between the Gd and the perfluorocarbon core of the DTPA complexes (**14** and **15**) vs. the Gd-DOTA chelate (**16**) further increased the fluorine relaxation rates (Hu et al., 2011). Subsequent work by de Vries et al. compared assemblies comprising DOTA-based Gd^III^ complexes to those containing Gd^III^ DTPA analogs (de Vries et al., 2014). Interestingly, Gd-DTPA-BSA (6,9-bis(carboxymethyl)-3-(2-(octadecylamino)-2-oxoethyl)-11-oxo-3,6,9,12-tetraazatriacontanoic acid) (**17**, *R*_1_ = 1.45 s^−1^) had a greater impact on the relaxation rates of the fluorines than Gd-DOTA-DSPE (2,2′,2″-(10-(2-((2-((((R)-2,3-bis(palmitoyloxy)propoxy)(hydroxy)phosphoryl)oxy)ethyl)amino)-2-oxoethyl)-1,4,7,10-tetraazacyclododecane-1,4,7-triyl)triacetic acid)(**18**,*R*_1_ = 1.05 s^−1^)and Gd-DOTA-C6-DSPE(2,2′,2″-(10-(2-((6-((2-((((R)-2,3-bis(palmitoyloxy)propoxy)(hydroxy)phosphoryl)oxy)-ethyl)amino)-6-oxohexyl)amino)-2-oxoethyl)-1,4,7,10-tetraazacyclododecane-1,4,7-triyl)triaceticacid)(**19**, Figure 2). This difference was attributed to a shorter ^19^F–Gd distance in the emulsions containing the Gd-DTPA complex. As predicted from the Bloch-Wangsness-Redfield theory, Gd^III^ has a limited effect on *R*_1_ of nearby fluorines above 6 T. This highlights the limitations of paramagnetic complexes at high field (Neubauer et al., 2008; Hu et al., 2011; de Vries et al., 2014).

These previous examples positioned the paramagnetic metal ions on the surface of the nanoparticles and, thus, a significant distance away from the ^19^F nuclei. To resolve this issue Kislukhin et al. designed nanoparticles that incorporated the paramagnetic metal ions directly into the fluorous core, thereby decreasing the ^19^F–M distance (Kislukhin et al., 2016). Despite the electronegativity of the fluorine atoms that reduce the basicity of the ligand, fluorinated β-diketone ligands form neutral, hydrophobic complexes that are sufficiently stable and dispersible in the perfluorocarbon core (**20**, Figure 2). Three different paramagnetic metal ions were evaluated: Fe^III^, Mn^II^, and Gd^III^. Of these, Fe^III^ had the greater impact on the *R*_1_ of the ^19^F nuclei, increasing it 13-fold, and the sensitivity of the nanoparticles, increasing it 5-fold. In comparison, Mn^II^ and Gd^III^ increased *R*_1_ 7- and 5-fold, respectively. Both Mn^II^ and Gd^III^ induced severe line broadening which decreased the sensitivity of the assembly. Advantageously, these nanoparticles are readily taken up by glioma cells. Since the nanoparticles also have low cytotoxicity (they reduce the viability of glioma cells by only 20%), they can be used to track cells *in vivo* 24 h post injection in mice. Do note, though, that it is not possible to distinguish between live and dead cells labeled with fluorine probes 24 h post injection, especially if the cells are engrafted subcutaneously.

#### Dependence on the magnetic field strength

**Figure 3:**
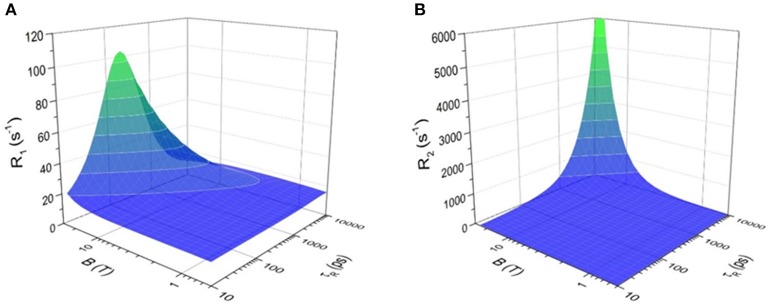
Effect of applied magnetic field, *B*, and the rotational correlation time, τ_R_ on **(A)** longitudinal relaxation rate (*R*_1_) and **(B)** transverse relaxation rate (*R*_2_) of ^19^F nuclei in [Tm-DOTAm-F12]^3+^. The analysis is based on Equation (1) for *R*_1_ or (2) for *R*_2_ and is done at 37°C using the mean ^19^F–Tm^III^ distance determined from X-ray crystallography (6.26 Å), assuming a magnetic moment, μ_eff_, of 7.6 BM and an electronic relaxation time, τ_e_, of 0.20 ps, values typical of Tm^III^ complexes.

As the Bloch-Wangsness-Redfield theory indicates, both the *R*_1_ and *R*_2_ of fluorine nuclei depend on the strength of the magnetic field (Figures 3A,B, respectively). The magnetic field dependencies of *R*_1_ and *R*_2_ are also a function of the μ_eff_ of the metal ion and as such, different metal ions are better suited for either low field or high field experiments. A steeper increase in *R*_1_ with respect to magnetic field strength is observed for Dy^III^ and Ho^III^ complexes of para-hydroxyl derivative of **4** than for its Tm^III^ and Tb^III^, and Er^III^ complexes (Chalmers et al., 2010; Harvey et al., 2012b). For this ligand scaffold, the stronger relaxing ions such as Ho^III^ are thus more appropriate for experiments performed at lower magnetic field strengths, whereas the weaker Tm^III^ and Er^III^ provide adequate relaxation enhancement above 7 T (Chalmers et al., 2010; Harvey et al., 2012b).

The higher relaxation rates of fluorine nuclei of paramagnetic complexes allow for the collection of more data per unit time. Consequently, PRE increases both the spectral sensitivity and the SNR of paramagnetic fluorine agents compared to their diamagnetic analogs. Chalmers et al. demonstrated that the Dy^III^ of fluorinated DOPA complex (**7**) increased the image SNR ~ 13-fold compared to the diamagnetic Y^III^ analog (Chalmers et al., 2011a,b). The improved SNR correlates with a *T*_2_/*T*_1_ ratio closer to l. With such a ratio, the benefit of increased *R*_1_ is not diminished by the increased signal broadening due to rapid transverse relaxation. Similar conclusions were drawn with complexes of DOTAm-F12 (Srivastava et al., 2017b). In theory, sensitivity gains of 15–20 times that of diamagnetic analogs can be achieved with fluorinated lanthanide complexes between 1.5 and 9.4 T, the magnetic fields of clinical relevance (Harvey et al., 2012b). The use of paramagnetic metal ions is thus the most effective strategy to increase the sensitivity of probes for ^19^F MRS/MRI, enabling imaging of sub-millimolar concentrations of probe in <15 min with adequate SNR (>4) (Chalmers et al., 2011a,b).

#### The effect of the rotational correlation time, τ_R_

Both the *R*_1_ and *R*_2_ of fluorine nuclei are affected by their rotational correlation time (τ_R_) as well as by the field strength of the instrument (*B*_0_) and the temperature of the sample. Both *R*_1_ and *R*_2_ increase as the magnetic field strength (*B*_0_) increases. This increase is far more significant if the rotational correlation time, τ_R_, is also optimized. Macromolecules, which tumble more slowly (longer τ_R_), have higher *R*_2_. As can be seen in Figure 3B, *R*_2_ increases drastically for ^19^F nuclei with τ_R_ > 1,000 ps, especially at high magnetic field (>7 T). The resulting extreme line broadening explains why fluorines embedded in bones and teeth in the body are not visible by MRI. The effect of τ_R_ on *R*_1_ is more complicated (Figure 3A). *R*_1_ increases to a maximum when the tumbling rate of the M-^19^F vector ($\tau_{\text{R}}^{-1}$) equals the Larmor frequency of the ^19^F nuclei; i.e., when $\omega_{F}^{2}\tau_{\text{R}}^{2}$ = 1 (Modo and Bulte, 2007). Monomolecular lanthanide complexes, such as those discussed in this review, have rotational correlation times on the order of 200–350 ps (Chalmers et al., 2010; Harvey et al., 2012b). Unlike for *R*_2_, there is an optimal τ_R_ beyond which *R*_1_ decreases sharply especially at high magnetic field. The optimal τ_R_ is a function of the μ_eff_ of the paramagnetic metal ion. For Tm, an optimal τ_R_ of 200 ps increases *R*_1_ substantially, particularly at high magnetic field. Unlike for Gd^III^-based *T*_1_ contrast agents (Caravan et al., 1999), there is not much advantage to macromolecular paramagnetic fluorine probes with longer τ_R_.

**Figure 4:**
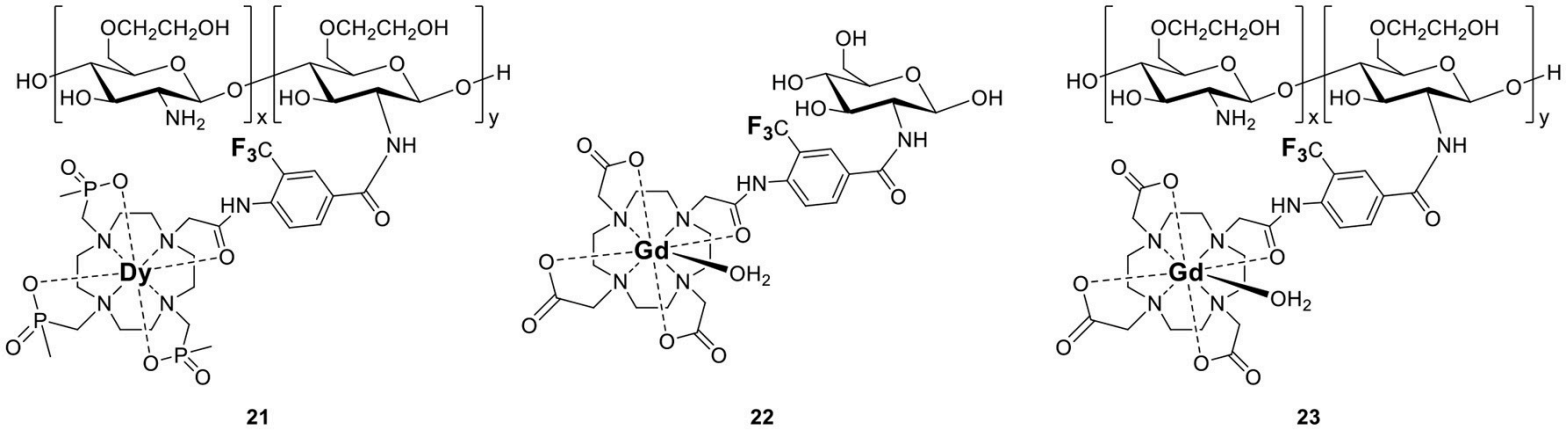
Non-responsive polymeric paramagnetic fluorine probes (**21–23**, De Luca et al., 2014).

There are limited examples in the literature of macromolecular fluorinated probes that feature a paramagnetic center. They are mostly used to increase the number of ^19^F nuclei per unit. For example, the polymeric Dy^III^-chitosan conjugate, **21** (Figure 4), increases the relaxation rates of the fluorines via the PRE effect (De Luca et al., 2014). As for the small molecules described earlier in this section, the aryl trifluoro groups are advantageously positioned close, but not too close (6.5 Å) from the lanthanide ion. This macromolecular complex features 10 trifluoro groups of equivalent ^19^F nuclei appended to glycol chitosan, a linear polysaccharide selected for its biocompatibility. The increase in τ_R_ from 83 ps to 8 ns due to polymerization of the chitosan was evaluated with Gd^III^-complexes similarly conjugated to the chitosan monomer (**22**) and polymer (**23**) (De Luca et al., 2014). In this case, the *R*_1_ of the fluorine nuclei of the Dy chitosan conjugate **21** remained constant before and after polymerization (186 s^−1^ at 9.4 T), whereas the *R*_2_ decreased from 440 to 367 s^−1^, which is unusual for a polymer (De Luca et al., 2014). Of note, this polymer is a rare example of a metallic fluorine probe that was successfully used *in vivo* to image tumors in mice. Although a dose of 0.54 g/kg was used in this study—a dose higher than typically used with Gd-based contrast agents—the sensitivity of the probe remained poor and a very long imaging time of 7 h was required to have sufficient SNR (De Luca et al., 2014). Although this probe was more sensitive than small molecule fluorine probes, the very long imaging time remains a significant hurdle to the application of ^19^F probes.

One should note that although macromolecular paramagnetic fluorine probes are rare, their diamagnetic analogs are more common. Several macromolecular structures based on hyperbranched polymers, dendrimers, or nanoparticles that incorporate a high number of ^19^F nuclei have been described (Du et al., 2008; Criscione et al., 2009; Jiang et al., 2009; Peng et al., 2009; Ogawa et al., 2010a,b; Thurecht et al., 2010), and recently reviewed (Yu, 2013; Zhu et al., 2013). A macromolecular architecture is more advantageous for diamagnetic fluorine probes than for paramagnetic ones: the increase in τ_R_ decreases *T*_1_, and, if designed correctly, can increase the solubility of the probe in water as well as its *in vivo* retention time.

## Exploiting the metal ion to design responsive fluorine probes

### Applications of the bloch-wangsness-redfield theory to the design of responsive fluorine probes

Advantageously, a paramagnetic metal ion that alters *T*_1_ and *T*_2_ of the fluorine nuclei can also modulate their chemical shift (^19^FΔδ). Since variations in each of these three variables can readily be observed both by MRS and by MRI, paramagnetic fluorine complexes offer unique mechanisms for the development of responsive MR probes that often yield much greater response than their purely organic analogs (Yu et al., 2013a). Two different effects of paramagnetic metals can be exploited to design responsive molecular probes. The first is the PRE effect. It is governed by the Bloch-Wangsness-Redfield theory, affects both relaxation times (*T*_1_ and *T*_2_) and is a function primarily of the nature of the paramagnetic metal ion and the distance *d* separating it from the fluorine nuclei. The second is the lanthanide induced shift (LIS) effect. It is just as readily be observed with ^19^F than with ^1^H nuclei and is governed by the McConnell-Robertson theory. Unlike PRE, LIS is affected not only by the nature of the paramagnetic lanthanide ion and the distance *d* separating it from the fluorine nuclei, but also by the second order crystal field coefficient of the complex, $B_{2}^{0}$, and the angle θ between the metal-fluorine vector and the principle magnetic dipolar axis of the lanthanide. Coupling either of these effects with the extensive range of chemical shifts available to ^19^F offers valuable opportunity to design ratiometric responsive MR probes that can distinguish between changes in signal intensity due to the presence of an analyte vs. that due to a change in the concentration of the probe.

The PRE effect described in the previous section of this review is the basis for the improved sensitivity on a per ^19^F basis of paramagnetic probes as compared to their organic counterparts. It is the substantial shorter *T*_1_ and higher *T*_2_/*T*_1_ ratio of paramagnetic fluorinated complexes that enables more scans to be acquired in the same amount of time thereby yielding higher SNR both by ^19^F MRS and MRI. Analysis of the Bloch-Wangsness-Redfield Equations (1–5) indicate that at constant applied magnetic field (the same scanner) and for the same metal ion, *T*_1_ and *T*_2_ are a function of primarily one parameter: the distance *d* separating the ^19^F nuclei from the metal ion. This parameter can be altered significantly by binding to or reacting with a substrate. Herein lies a substantial advantage of paramagnetic metal ions in fluorine probes: they readily enable the design of responsive and ratiometric molecular probes.

**Figure 5:**
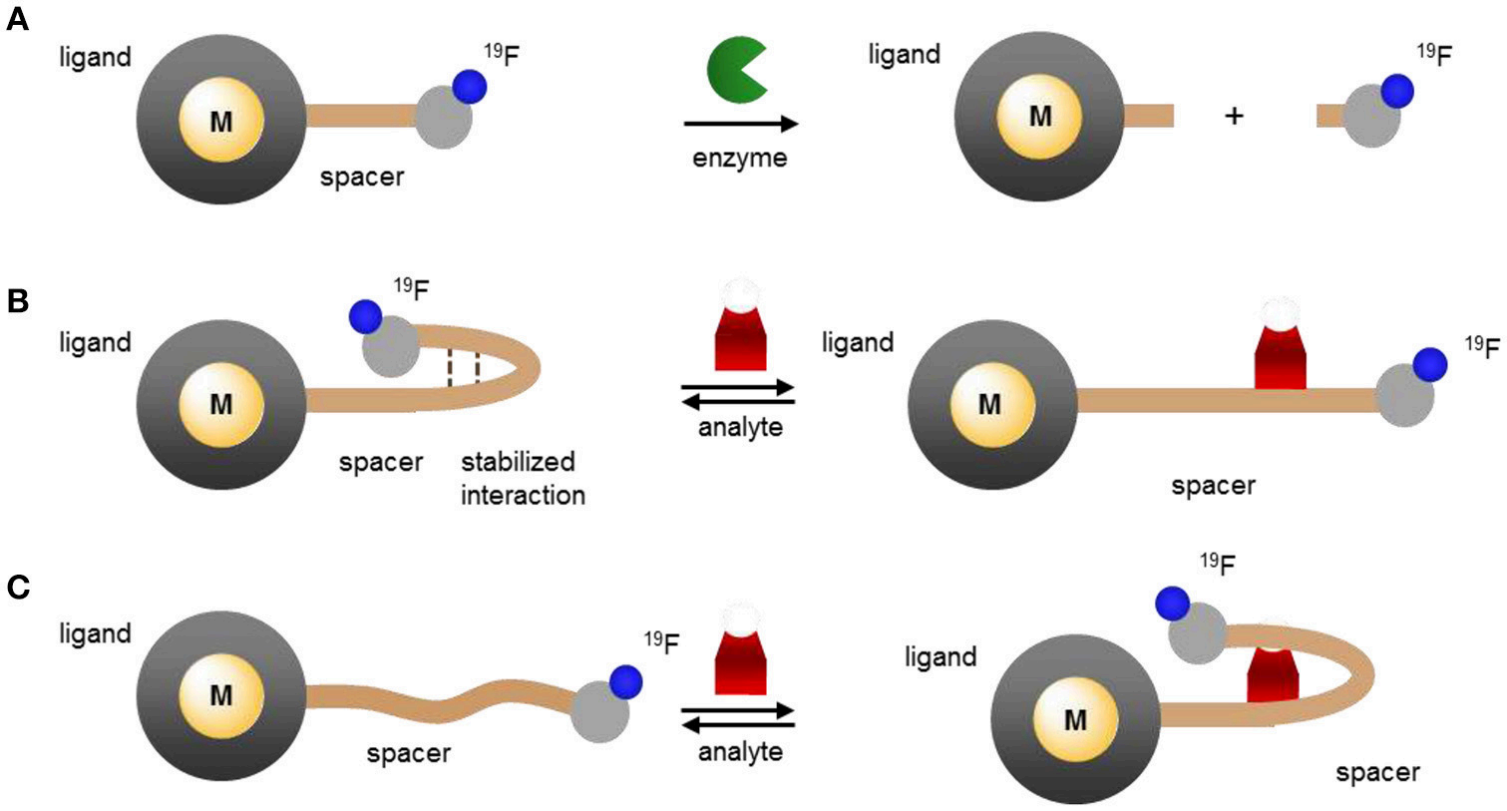
Modulating the response of ^19^F-paramagnetic metal probes by altering the distance separating the lanthanide ion from the ^19^F nuclei: **(A)** cleavage of the ^19^F-M distance spacer by the analyte and release of the fluorinated moiety; **(B,C)** analyte-triggered conformational change of the probe upon binding or reacting with the spacer.

The dependence of *T*_1_ and *T*_2_ on the distance separating the fluorine nuclei from the lanthanide ion has already been applied to the design of responsive paramagnetic fluorine probes. As depicted in Figure 5, this distance can be altered either by reacting with a target or by induced conformational change upon binding to the desired analyte. The former approach yields an irreversible response, although one that can enable the detection of targets such as enzymes that are present at lower concentrations. The latter approach can lead to a reversible response but given the enduring limits in the sensitivity of fluorine probes, it is more appropriate for biological analytes present at substantially higher concentrations such as pH or certain small molecules or metal ions.

Current examples of this class of responsive probes use for the most part Gd^III^. As described in the previous section, Gd^III^ has a notable effect on the *T*_1_ of nearby nuclei, and, importantly, a substantially more pronounced one on *T*_2_. Indeed, among the lanthanides, Gd^III^ typically offers the lowest *T*_2_/*T*_1_ ratio (Table 2). As a result of this very low ratio, the peaks of ^19^F nuclei near Gd^III^ ions broaden to the point of no longer being observable neither by MRS nor by MRI. Increasing the distance between the ^19^F nuclei and the Gd^III^ increases *T*_2_; this enables the ^19^F signal to reappear.

**Figure 6:**
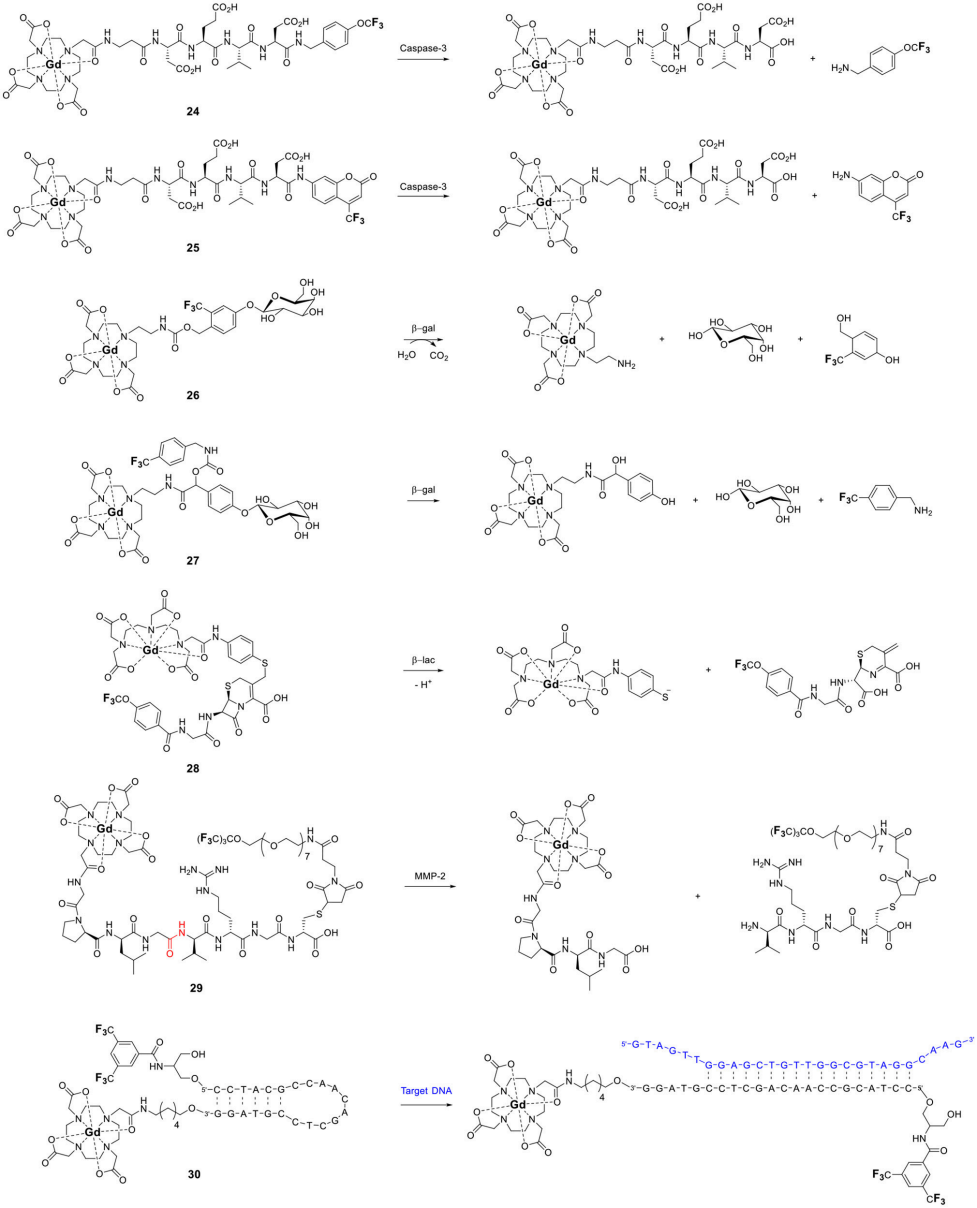
Responsive fluorinated probes whose signals are modulated by the paramagnetic relaxation enhancement effect of Gd^III^ (**24**, Mizukami et al., 2008; **25**, Mizukami et al., 2009; **26**, Mizukami et al., 2011; **27**, Keliris et al., 2012; **28**, Matsushita et al., 2012; **29**, Yue et al., 2014, 2017; **30**, Sakamoto et al., 2011).

A greater effect will be observed if the linker separating the ^19^F nuclei from the Gd^III^ ion is completely cleaved by the target (Figure 5A). As the fluorinated moiety diffuses away from the Gd^III^ complex, its relaxation times increase by orders of magnitude and, consequently, so does the ^19^F signal intensity. This approach is particularly well-suited for the detection of enzymes that cleave peptides or sugars. Indeed, the first example reported by Kikuchi and coworkers targeted caspase 3 (**24**, Figure 6). Adroit positioning of the ^19^F nuclei on the amino acid substrate of the enzyme that was directly tethered to the Gd^III^ complex resulted in a complete off/on response (Mizukami et al., 2008). This first probe, and its dual ^19^F MRI/fluorescence analog (**25**, Figure 6) (Mizukami et al., 2009), exemplified a distinct advantage of ^19^F molecular probes over both Gd^III^- and nanoparticle-based responsive contrast agents: a substantial response with zero background such that presence of a signal can only result from co-localization of both the MR probe and the enzyme target. In comparison, ^1^H MRI contrast agents have to contend with the intrinsic high ^1^H background signal due to the high concentration of water of the biological milieu. With these agents, an increase in signal intensity does not necessarily correlate with the presence of the target; an identical response can simply result from higher local concentration of the contrast agent. Do note, however, that since such agents are not ratiometric they do not eliminate the possibility of false negative. That is, they cannot distinguish between a lack of signal due to a lack of enzyme as opposed to a lack of ^19^F probe.

This approach was recently extended by both the Kikuchi and the Engelmann groups to other enzymes, β-galactosidase (**26** and **27**) (Keliris et al., 2011, 2012; Mizukami et al., 2011), β-lactamase (**28**) (Matsushita et al., 2012), and matrix metalloprotease-2 (**29**, Figure 6; Yue et al., 2014, 2017) thereby opening the possibility of directly imaging gene expression by MRI. In each case, hydrolysis of the enzyme's substrate generates a self-immolative linker that releases the fluorinated moiety from the Gd^III^ probe thereby turning on the ^19^F MR signal. This approach is not limited to enzymes and cleavable linkers. Similar responses can be obtained with an analyte-induced conformational changes that either increase (turn on) or decrease (turn off) the ^19^F–Gd distance (Figure 5B,C, respectively). Fujimoto and coworkers used this approach to the PRE effect to extend the traditional molecular beacon motif commonly used to detect DNA (or RNA) by luminescence to that by ^19^F NMR (**30**, Figure 6) (Sakamoto et al., 2011). Hybridization with the target nucleotide sequence causes the stem loop of the probe to unfold, thereby increasing *d* and thus also the *T*_2_ and signal intensity of the ^19^F nuclei.

**Figure 7:**
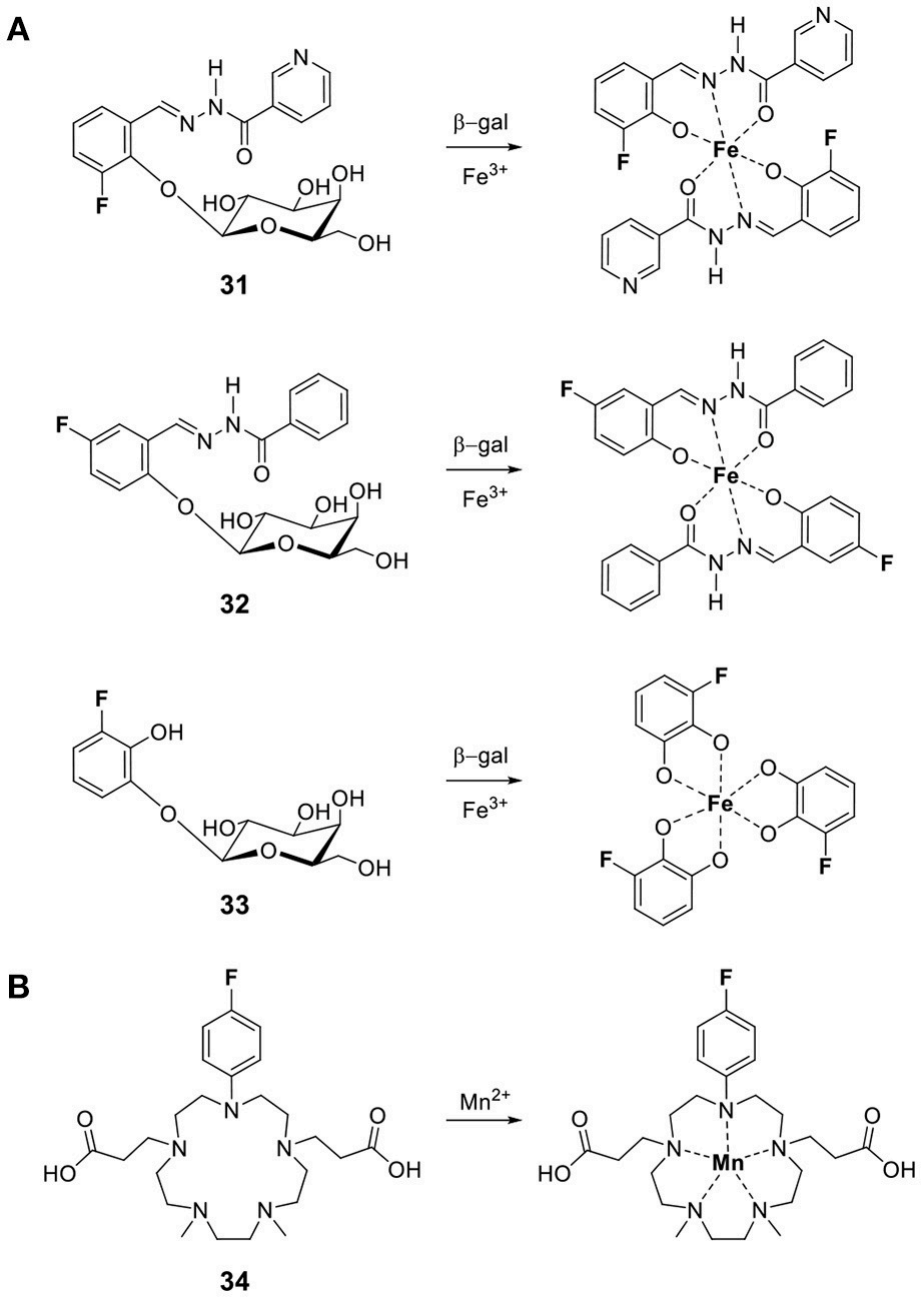
Responsive fluorine probes whose signals are modulated via the paramagnetic relaxation enhancement effect upon complexation of **(A)** Fe^III^ following cleavage by β-galactosidase (**31**, Yu et al., 2012; **32–33**, Yu et al., 2013b) or **(B)** Mn^II^ ions (**34**, Sarkar et al., 2017).

Similar responses can be obtained with paramagnetic transition metals. Yu and Mason reported three fluorinated probes for β-galactosidase that use the same principle but in the opposite direction (**31–33**, Figure 7) (Yu et al., 2012, 2013b). Hydrolysis of the sugar moiety by β-galactosidase releases a fluorinated Fe^III^ chelator. The *T*_2_ of this ligand decreases substantially upon subsequent coordination to the transition metal. As opposed to Kikuchi's and Engelman's examples, in this case the ^19^F signal of the probe is turned off by the enzyme. The practicality of such probes for *in vivo* imaging, however, is likely limited given that iron is tightly regulated in the body and is essentially unavailable (Raymond et al., 2003).

The same approach readily leads to the development of fluorine probes for paramagnetic metal ions. An example for Mn^II^ is **34** developed by Datta and coworkers (Figure 7B) (Sarkar et al., 2017). Coordination of Mn^II^ results in a 101-fold decrease of *T*_2_ and, therefore, significant line broadening which decreases the signal intensity of the ^19^F nuclei.

**Figure 8:**
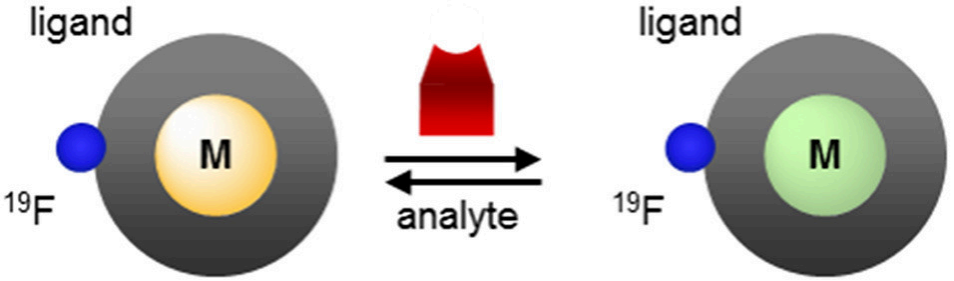
Modulating the response of ^19^F-paramagnetic metal probes by altering the effective magnetic moment of the metal ion.

**Figure 9:**
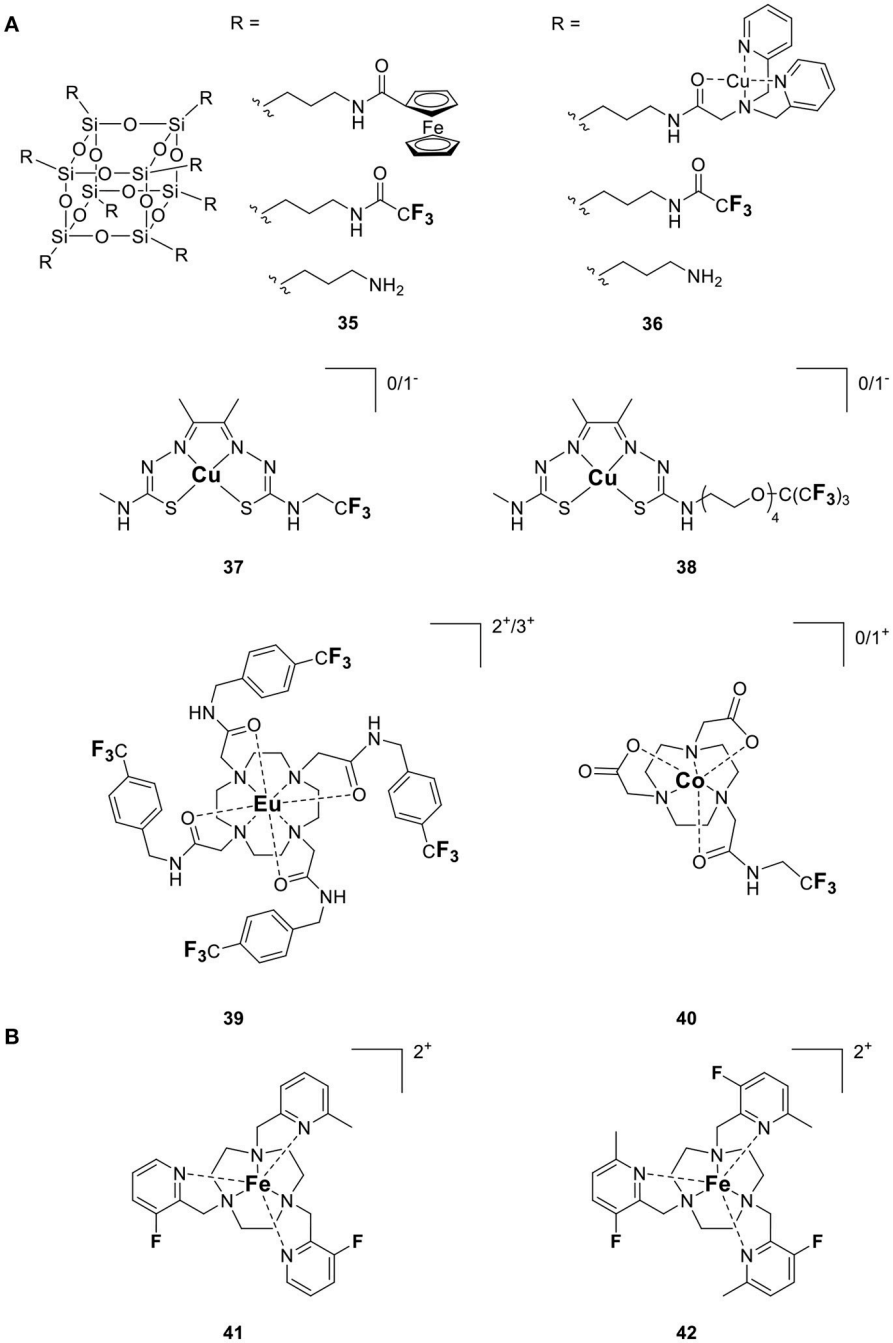
Responsive fluorine probes which function by altering either **(A)** the oxidation state of a redox active metal (**35**, Tanaka et al., 2009; **36**, Kitamura et al., 2012; **37**, Xie et al., 2016; **38**, Xie et al., 2017; **39**, Basal et al., 2017; **40**, Yu et al., 2016) or **(B)** the spin state of Fe^II^ (**41–42**, Thorarinsdottir et al., 2017).

By extension, this strategy can readily be adjusted to design probes that signal the redox potential of their surroundings. If the metal is chosen correctly, a change in its oxidation state enables it to switch between a diamagnetic and a paramagnetic state (Figure 8). In the paramagnetic state, the metal attenuates the ^19^F signal by substantially decreasing their *T*_2_. Conversion to the diamagnetic state lengthens *T*_2_ resulting in a “turn on” response. Early examples that utilize metal redox state to modulate the relaxation of ^19^F nuclei, and in turn signal intensity, were reported by Chujo's lab (Tanaka et al., 2009; Kitamura et al., 2012). Their probes, Fc-POSS (**35**) and Cu-POSS (**36**) contain either a redox sensitive ferrocene or a Cu complex with a bis(2-pyridylmethyl)amino ligand (PMEA) along with trifluoromethyl groups appended to a cubic polyhedral oligomeric silsesquioxane (POSS) for water solubility (Figure 9A). The oxidation of Fe^II^ in **35** reduces the ^19^F signal intensity 3-fold due to the shortened relaxation time. The nitroxyl (HNO) mediated reduction of paramagnetic Cu^II^ in **36** to diamagnetic Cu^I^ modestly increased *T*_1_ (from 1180 ms to 1460 ms) and *T*_2_ (from 420 to 540 ms) causing a 3-fold increase in signal intensity. Thus, their work demonstrates the ability to apply PRE to the design of ^19^F redox sensitive responsive probes. Que's laboratory utilized this strategy and the PRE of Cu^II^ to detect cellular hypoxia by ^19^F MR (Xie et al., 2016). Reduction of CuATSM-F_3_ (**37**, Figure 9A) from Cu^II^ (d^9^, paramagnetic) to Cu^I^ (d^10^, diamagnetic) and subsequent ligand dissociation increases *T*_2_ of the ^19^F and restores their signal intensity. A second generation version of this probe (**38**) was designed to be more sensitive (Xie et al., 2017). It includes a higher density of ^19^F nuclei, a polyethylene glycol linker that increases the hydrophilicity of the probe and positions the ^19^F nuclei at an optimal distance from the copper ion. With a ^19^F–Cu distance of ~ 18 Å, the ^19^F signal is attenuated but remains visible by NMR when the probe is in the Cu^II^ state. Reduction to Cu^I^ generates a sharp signal which is 6-fold more intense, leading to a 4.5-fold increase in SNR in the ^19^F MRI images obtained at 7 T. The large differences in relaxation times between the two species allow them to be independently imaged using short (TE = 10 ms for Cu^II^) or long (TE = 100 ms for Cu^I^) echo pulse sequences (Xie et al., 2017).

Allen's laboratory used an analogous approach with Eu in the design of their temperature and redox responsive ^19^F probe (**39**, Figure 9A). This Eu-DOTAm (1,4,7,10-tetrakis(carbamoylmethyl)-1,4,7,10-tetraazacyclododecane) complex with a *p*-trifluoromethylphenyl arm, has 12 equivalent fluorine nuclei (Basal et al., 2017). In its +2 oxidation state, Eu significantly shortens the *T*_2_ of the ^19^F and hence quenches their signal. In its +3 oxidation state, Eu^III^ has a much lower μ_eff_ (3.40–3.51 B.M.) than Eu^II^ (7.6–8.0 B.M.; Tilley et al., 1980; Garcia and Allen, 2012) (Table 1) and thus a limited effect on the relaxation times of the fluorine which consequently reappear. In ^19^F MRI images, oxidation of Eu^II^ to Eu^III^ results in a 6-fold increase in SNR. This responsive probe was successfully used to image non-hypoxic regions *in vivo* by ^19^F MRI.

This approach also enables direct detection of reactive oxygen species. Oxidation of Co-NODA-CF_3_ (2,2′-(7-(2-oxo-2-((2,2,2-trifluoroethyl)amino)ethyl)-1,4,7-triazonane-1,4-diyl)diacetic acid) (**40**, Figure 9A) with H_2_O_2_ from paramagnetic Co^II^ (d^7^) to diamagnetic Co^III^ (d^6^) shifts the ^19^F signal by 3.6 ppm and increases the *T*_1_ and *T*_2_ of the ^19^F nuclei 60- and 121-fold, respectively (Yu et al., 2016). This, in turn, results in a 23-fold increase in signal intensity in spin-density weighted ^19^F MR images. Although the oxidation of the probe is reversible, the long (2 h) reaction time and the low sensitivity of the probe compared to *in vivo* levels of H_2_O_2_ still limit the application of this probe to *in vivo* imaging.

There are two main disadvantages to using paramagnetic metals that primarily affect *T*_2_ in the design of responsive fluorine probes. The first, as mentioned above, is that such probes are not ratiometric. A lack of signal can be attributed both to a lack of target and to a lack of probe. Similarly, signal intensity is not a direct measure of the concentration of the target as it is equally affected by the concentration of the probe. The second disadvantage is that upon release, the organic fluorine moiety regains its very long *T*_1_. Such probes are thus plagued by the same sensitivity issue as the purely organic fluorinated probes (Yu et al., 2005, 2013a). Their *T*_1_ of 0.4–5 s are10- to 500-fold longer than their paramagnetic counterparts, which greatly limits the number of scans that can be acquired per amount of time. The low sensitivity of such probes practically restricts the markers that they could measure *in vivo*. In theory, both of these problems can be resolved with the use of a paramagnetic ion that has a more limited effect on *T*_2_ and maintain as high a *T*_2_/*T*_1_ ratio as possible. Fe^II^, Tm^III^, and Ho^III^ are particularly well-suited for this approach (Srivastava et al., 2017b), but examples are not represented in the literature.

### Applications of the mcconnell-robertson theory to the design of responsive fluorine probes

One of the first applications of lanthanides in NMR spectroscopy was as induced shift reagents (Sanders et al., 1972). This application has surprisingly rarely been applied to ^19^F NMR spectroscopy. Yet the same theory and equations developed for ^1^H apply to ^19^F and as such they can readily be exploited for the rational design of paramagnetic ^19^F shift reagents. Lanthanide induced shift (LIS) is traditionally defined as the difference in chemical shift, Δδ, between a paramagnetic complex and its diamagnetic analog (Δδ = δ_para_ – δ_dia_). In terms of responsive paramagnetic fluorine probes, the induced shift, ^19^FLIS, is defined as the difference between the chemical shift of a ^19^F nuclei in the presence and absence of the probe's analyte (Equation 6).

(6) $${}^{19}FLIS{=}^{19}\text{​​}F\Delta \delta =\delta_{\text{probe }+\text{ analyte}}\;-\;\delta_{\text{probe}}$$

**Figure 10:**
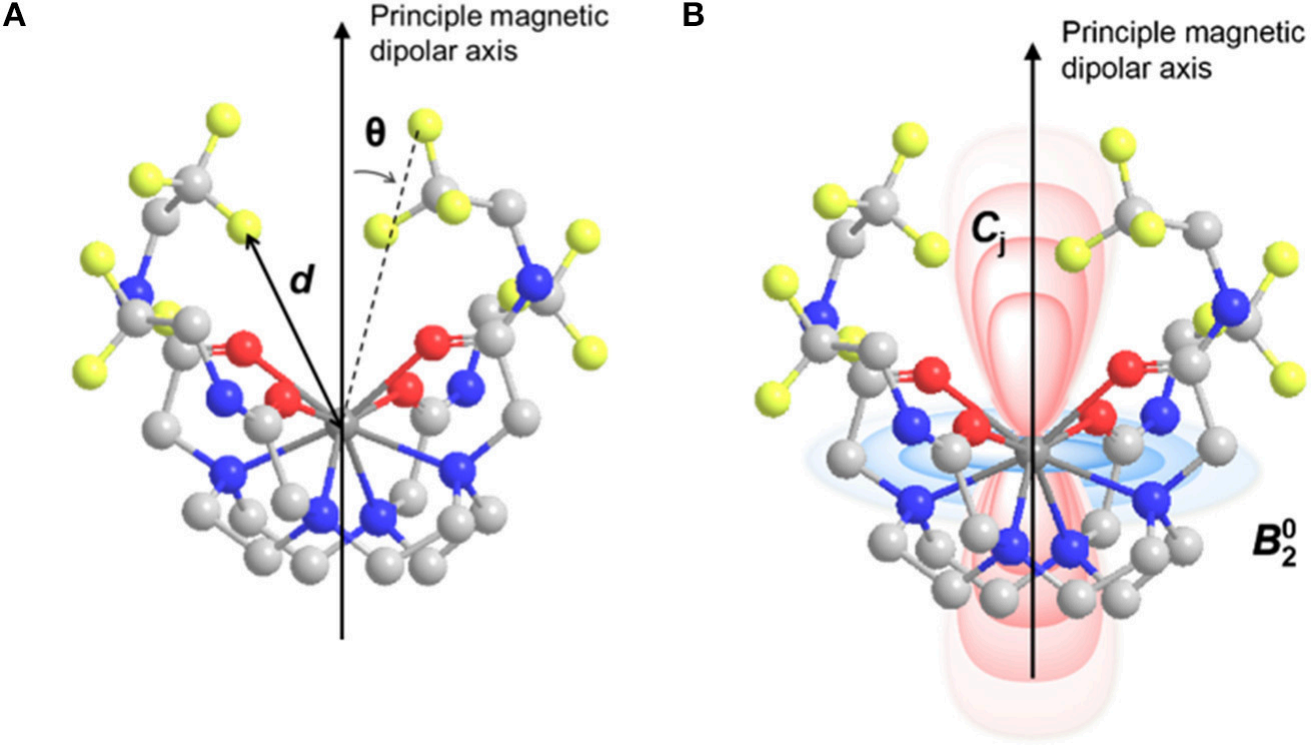
Model of M-DOTAm-F12 illustrating **(A)** the distance, *d*, between the ^19^F nuclei and the metal ion and the angle, θ, between the ^19^F–M vector and the principle magnetic dipolar axis of the metal ion; and **(B)** the positive (red) and negative (blue) regions of the pseudocontact shift field, $B_{2}^{0}$, surrounding the metal ion.

This shift is the sum of the contributions from the contact shift, δ^c^, and the pseudocontact shift, δ^pc^ (Peters et al., 1996; Allegrozzi et al., 2000; Harvey et al., 2012b). If the fluorine nuclei is kept 5 Å or more from the paramagnetic metal center, the contribution from the contact shift is negligible, and the observed ^19^FLIS is primarily due to the pseudocontact shift. This pseudocontact shift, δ^pc^, is the result of the dipolar interaction between the ^19^F nuclei and the unpaired electrons on the metal ion that experiences a Curie magnetization in the applied magnetic field. Thus, an anisotropic metal ion can amplify the chemical shift inequivalence between a fluorine nucleus in the presence and absence of the probe's analyte, resulting in greater resonance shift that are ultimately necessary for each nuclei to be imaged independently by MRI. Under the condition that the ^19^F nuclei is at least 5 Å away from the metal ion, the pseudocontact shift is a function of the nature of the metal, its coordination environment, and the metal ion (Figure 10). This relationship is defined by the McConnell-Robertson equation (Equation 7) (Harvey et al., 2012b)

(7)  F19LIS= δpc= Cj β260(kT)2(3cos2θ− 1)d3B20

Where *d* is the ^19^F–metal distance, θ is the angle between the principal magnetic dipolar axis of the metal ion and ^19^F nuclei, $B_{2}^{0}$ is the second order crystal field coefficient that is dependent on the coordination environment of the metal ion, and *C*_j_ is the Bleaney coefficient for the specific metal. Both the direction and magnitude of the shift are dependent on the identity of the metal ion as denoted by its Bleaney coefficient (Table 1). One can thus rank metals by relative pseudocontact shift (PCS) strength, which in turn indicates their efficacy as ^19^FLIS agents. Dy^III^ and Tb^III^ are undeniably excellent shift agents. However, given the necessity to maintain as high a *T*_2_/*T*_1_ ratio as possible to maximize ^19^F sensitivity (see above), Tm^III^ and Ho^III^ are the best choices in the design of ^19^FLIS probes for MRI.

**Figure 11:**
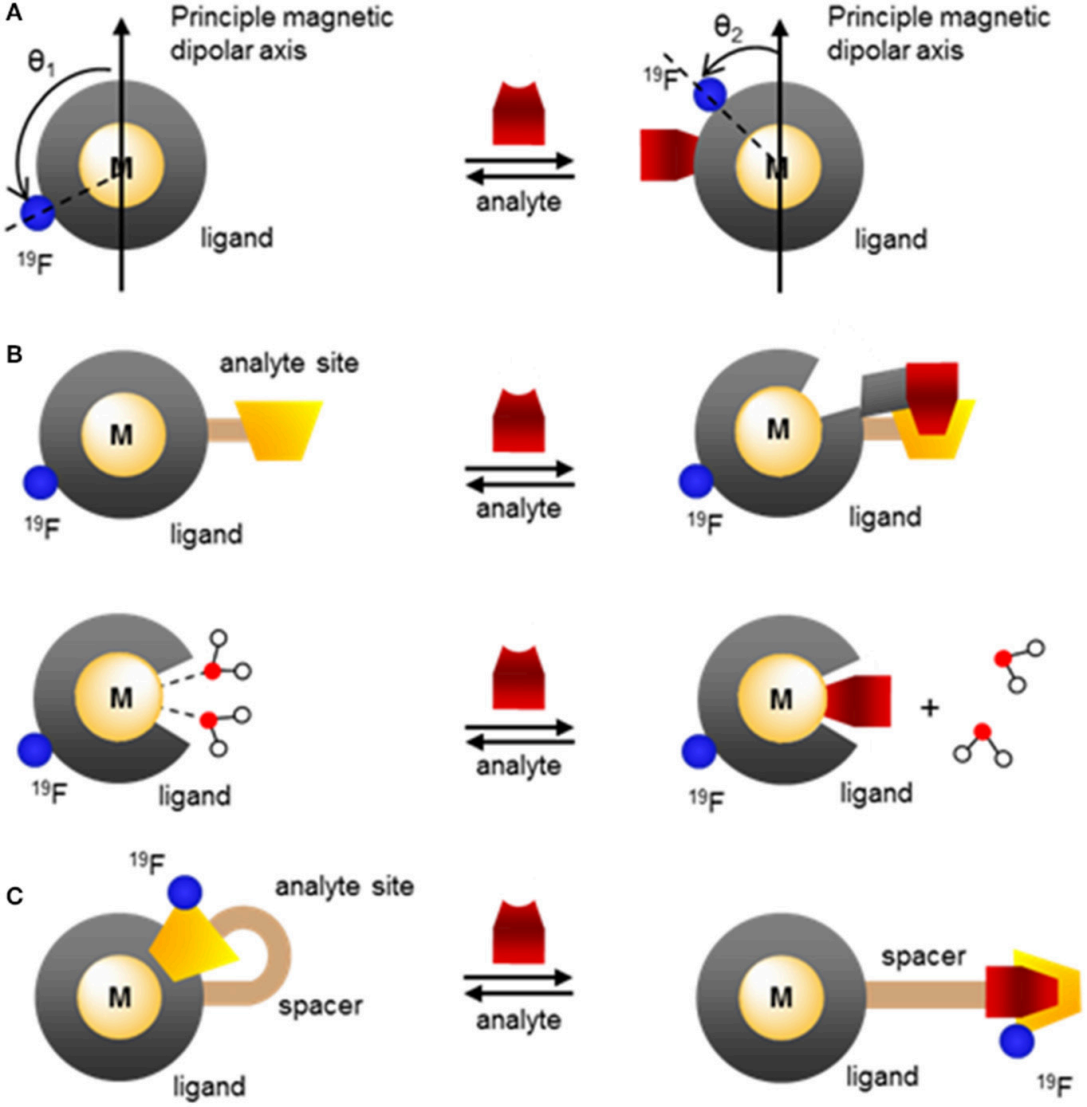
Modulating the response of fluorinated paramagnetic probes by altering **(A)** the angle θ between the ^19^F–M vector and the principle magnetic dipolar axis of the metal ion, **(B)** the lanthanide coordinating environment and thus the second order crystal field coefficient, $B_{2}^{0}$ through chelation of the analyte that partially decomplexes the metal or direct coordination of the analyte onto the lanthanide that displaces one or more water molecules, **(C)** the angle θ, $B_{2}^{0}$, and the ^19^F–M distance, *d*, through analyte induced changes in the coordination environment.

According to the McConnell-Robertson equation, the design of responsive fluorinated lanthanide MR probes can be based on three parameters: the distance separating the ^19^F nuclei from the metal ion, *d* (Figure 5), the angle θ between the main dipolar magnetic axis of the metal and the metal—^19^F vector (Figure 11A), and the second order crystal field coefficient, $B_{2}^{0}$, which is a function of the direct coordination environment of the metal (Figures 11B,C).

**Figure 12:**
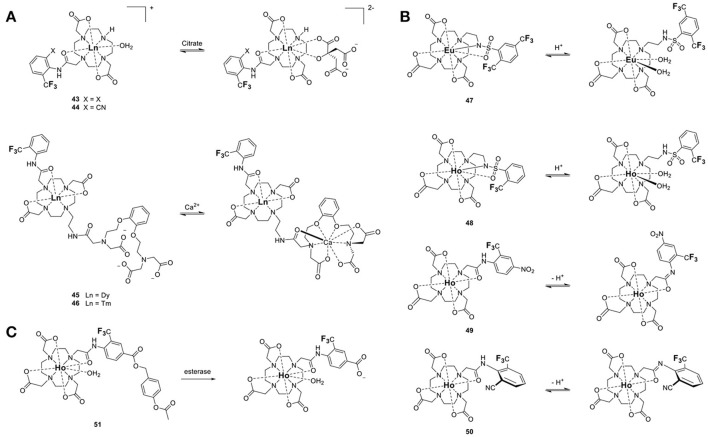
Responsive ^19^F-Ln probes that induce a chemical shift (^19^FΔδ) by **(A)** modulating the coordination environment of the Ln^III^ and hence its second order crystal field coefficient, $B_{2}^{0}$, (**43–44**, Harvey et al., 2012a; **45–46**, Harvey et al., 2012a), **(B)** altering $B_{2}^{0}$ and the angle θ between the Ln^III^-^19^F vector and the principle magnetic dipolar axis of the lanthanide upon changes in protonation (**47**, Senanayake et al., 2007; **48–49**, Kenwright et al., 2008; **50**, Chalmers et al., 2010), or **(C)** following a chemical reaction (**51**, Harvey et al., 2012a).

The second order crystal field coefficient, $B_{2}^{0}$, which is a function of the direct coordination environment of the lanthanide or transition metal, has been used in the design of responsive fluorine probes. Advantageously, this approach enables the use of ligands of similar chemical structure as those used with gadolinium-based responsive contrast agents. Swapping Gd^III^ for Tm^III^, Ho^III^, or Dy^III^ and adding a fluorinated moiety within 4–7 Å of the metal is often all that is necessary. For example, the Parker group developed citrate-responsive ^19^F probes, which are essentially fluorinated versions of Ln-DO3A (**43** and **44**, Figure 12A) (Harvey et al., 2012a). Coordination of citrate to a Tm^III^ complex results in the displacement of the two inner-sphere water molecules; the resulting change in $B_{2}^{0}$ causes a ^19^FLIS of 4.7 ppm. Such ^19^F probes are unfortunately marked with the same selectivity problem as the gadolinium responsive contrast agents that function by varying the number of inner-sphere water molecules (*q*-based responsive contrast agents) (Pierre et al., 2018). The selectivity of **43** and **44** for citrate over other hard anions that also have good affinity for the oxophilic lanthanides, such as bicarbonate, phosphate and lactate, is rather poor. The efficacy of the probe in blood and in cells is thus likely limited.

The same approach was used to design the Ca^II^ responsive fluorine probes **45** and **46** (Figure 12A). These probes are derivatives of a Gd^III^-based responsive contrast agent which displays a change in longitudinal proton relaxivity, *r*_1_, in the presence of calcium (Harvey et al., 2012a). For **45** and **46** to bind Ca^II^, one of the amide arms of the macrocycle must release the lanthanide. In doing so, it is replaced by a water molecule. The resulting change in $B_{2}^{0}$ causes a ^19^FLIS of 3.0 or 3.9 ppm depending on the lanthanide ion (Dy^III^ or Tm^III^, respectively) (Harvey et al., 2012a). Seemingly minor modifications on the structure of the probe can thus result in a substantial difference in its response.

The Parker group used a similar approach to develop four pH responsive probes. In **47** and **48** (Figure 12B) (Senanayake et al., 2007; Kenwright et al., 2008), protonation of a sulfonamide group coordinating the lanthanide at pH <5 breaks the N-Ln bond, which opens up a coordination site for a water molecule. The resulting change in $B_{2}^{0}$ due to the differing coordination environment of the lanthanide ion, and in the angle θ due to conformational change through rotation of the arm, cause a ^19^FLIS of −6 and +2 ppm between pH 8.5 and 4.2 for the two inequivalent ^19^F in the Eu^III^ complex **47**. The ^19^F resonance of the related Ho probe, **48**, shifts upfield by 40 ppm upon deprotonation (Chalmers et al., 2010). Note the greater ^19^FLIS for Ho^III^, which has a Bleany's coefficient of greater magnitude, than for Eu^III^.

The other two pH probes, **49** and **50**, also rely on protonation of a coordinating arm, but in these cases the aryl amide does not release the lanthanide at acidic pH (Figure 12B) (Kenwright et al., 2008; Chalmers et al., 2010). Instead, deprotonation of the amide changes both $B_{2}^{0}$ and θ between the Ln-^19^F vector and the principal magnetic dipolar axis of the Ln^III^ ion. The latter is due to the differing conformation of the arm. The combination of these two effects result in a substantial ^19^FLIS of 18 and 23 ppm for **49** and **50**, respectively.

The chemical shift of fluorine nuclei can also be modulated by altering the spin state of the metal ion. A recent example exploits the temperature induced transition of Fe^II^ from low spin (*S* = 0) to high spin (*S* = 2) in hexadentate triazacyclonone ligands with fluorinated 2-picolyl substituents (**41** and **42**, Figure 9B) to image temperature by ^19^F MRI (Thorarinsdottir et al., 2017). In these probes, the ^19^F are positioned at an optimal distance away from the Fe^II^ center so as to maximize ^19^FΔδ. Increasing the temperature from 4 to 60°C, causes the ^19^F resonances of **41** to shift downfield 28.3–24.6 ppm. The single resonance of **42** instead shifts 12 ppm upfield. On a per °C basis, **41** is therefore a more sensitive probe than **42**. Importantly, the response of these two probes to temperature is nearly identical in water and fetal bovine serum (FBS), which bodes well for future *in vivo* applications.

The standard approaches that are used in the design of diamagnetic fluorine probes can also be applied to their paramagnetic analogs. An example is the α-chymotrypsin esterase probe **51** (Figure 12C). Hydrolysis of the ester, followed by self-immolation of the linker, yields a carboxylated fluorine moiety whose ^19^F nuclei signal is positioned 6.2 ppm away from the starting material (Harvey et al., 2012a). These probes function essentially like their purely organic analogs recently reviewed by Mason (Yu et al., 2005). The advantage of the paramagnetic version resides in the sensitivity of the probe (see section Improving the Sensitivity of ^19^F Probes). In this case, the lanthanide ion functions only to increase the sensitivity of the probe. Ho^III^ shortens the *T*_1_ and *T*_2_ of the fluorine, thereby significantly increasing the number of scans that can be recorded in a given amount of time, and thus improving the SNR of the probe.

Although this review has focused on ^19^F MR imaging, a significant advantage of ^19^F probes is their ability to also function in other modalities. Fluorinated polyaminocarboxylate-based complexes, for instance, can also be effective chemical exchange saturation transfer (CEST) MR contrast agents. Such dual-modalities are particularly well-suited for the design of ratiometric agents. False negatives are more readily avoided if one of the two modalities is responsive to the targeted marker while the other one is not. This enables the distribution of the contrast agent to be mapped independently of that of the marker. Two examples of dual ^19^F-CEST agents have been reported. A fluorinated Eu-DOTAm-Gly complex with two *trans* trifluoromethyl groups (**2**, Figure 1) generated sufficient ^19^F and CEST contrast *in vitro* despite multiple isomers in solution (Cakic et al., 2016). Unfortunately, the contrast agent was not detected in *ex vivo* by ^19^F MRI, possibly due to interactions with tissues that substantially reduced *T*_2._ This first dual-modality ^19^F contrast agent is not responsive.

Fe-DOTAm-F12 which was developed in our lab, has 12 equivalent fluorine nuclei and one isomer in solution (**12**, Figure 1). It functions both as a ^19^F MR probe and a paraCEST contrast agent. Importantly, whereas the ^19^F signal intensity is independent of pH, the CEST signal is modulated by pH. The % saturation transfer of the complexes increases 5-fold between pH 4 and 6.2. The complex accurately determines the pH independently of the concentration of the contrast agent between pH 6.9 and 7.4, a range that is relevant to cancer diagnosis. Advantageously, the similar sensitivity of the agent in both modalities facilitates ratiometric determination of pH.

## Outlook

The development of paramagnetic fluorine probes over the last decade mirrors, in many ways, that of paraCEST agents, the paramagnetic derivatives of CEST contrast agents. The introduction of paramagnetic metal ions in the design of fluorine probes, most notably lanthanides, iron, and cobalt, offer several advantages. Those include increased sensitivity of the fluorine probes due to decreased *R*_1_ of the fluorine nuclei and increased chemical shift spectral window due to the paramagnetic induced shift.

Advantageously, both the effects on relaxation rates and chemical shift offer new approaches to the development of responsive fluorine probes that are not available with their diamagnetic counterparts, and these strategies lead to a significantly greater response. Indeed, paramagnetic fluorinated complexes can be readily designed such that the chemical shifts and/or the relaxation times *T*_1_ and *T*_2_ of the fluorine nuclei change substantially upon binding to or reacting with a desired analyte. This causes either the appearance, disappearance or notable chemical shift of the ^19^F peaks. Moreover, the absence of background signal in ^19^F MRI is an added advantage of this class of probes over the more common Gd-based and iron oxide nanoparticle-based contrast agents which have to contend with the signal of endogenous water. The recent work published on paramagnetic fluorine probes, backed by accurate theoretical models, enable scientists to design molecules with maximum sensitivity by optimizing the nature, specially the oxidation and spin state, of the paramagnetic ion and its distance from the fluorine nuclei all while maximizing the number of chemically equivalent ^19^F nuclei. Those same parameters are used in the design and optimization of responsive probes. Notably, the large spectral windows of paramagnetic fluorine probes enable the design of ratiometric responsive probes which can independently report on both the distribution of a probe and that of its targeted analyte. Paramagnetic fluorine probes are also uniquely suited to multicolor, or multifrequency, imaging, which enables the tracking of different types of cells.

Paramagnetic fluorine probes have drawbacks too that should be kept in mind. The increase in sensitivity due to lower *R*_1_ induced by the paramagnetic ion is minimal at high magnetic fields. These probes are better suited for use with scanners of low and medium magnetic field strengths, below 6 T. The structure and conformation of the probe in solution, parameters that can be more difficult to predict, have a significant impact on the properties of the probe. Complexes existing as a single isomer in solution with all fluorine nuclei chemically equivalent are more sensitive and easier to image, but many lanthanide complexes exist in solution as interconverting isomers. The potential toxicity of the complex should also be kept in mind, especially given the high concentrations of probes required for *in vivo* imaging. Although the toxicity and pharmacological properties of this new class of probes has not yet been evaluated, conclusions drawn from Gd-based contrast agents are likely to extend to paramagnetic fluorine probes. Kinetically labile lanthanide complexes, for instance, are expected to present higher toxicity. Nonetheless, we foresee that these first generation of paramagnetic fluorine probes will open avenues for many more applications for *in vivo* imaging. In particular, the ability of these probes to track different cells and biomarkers, and their response to different stimuli, render them particularly promising for molecular imaging.

## Author contributions

VP, KP, and KS: searched and analyzed the literature; VP, KP, and KS: co-wrote the manuscript.

### Conflict of interest statement

The authors declare that the research was conducted in the absence of any commercial or financial relationships that could be construed as a potential conflict of interest.
